# Bioactive spectrum of goji berries (*Lycium* spp.) and their functional applications in food systems: A comprehensive review

**DOI:** 10.1016/j.fochx.2026.104253

**Published:** 2026-07-31

**Authors:** Muthu Thiruvengadam, Hee-Youn Chi, Ja-Min Lee, Yunwoo Park, Dagyeom Jeon, Seung-Bin Lee, Yong-Ik Jin, Hae Won Jang, Rekha Thiruvengadam, Baskar Venkidasamy, Seung-Hyun Kim

**Affiliations:** a Department of Crop Science, College of Life Science, Konkuk University, Seoul 05029, Republic of Korea; b R&D Planning Division, Research Policy Bureau, RDA, Jeonju-si 54875, Republic of Korea; c Department of Food Science and Biotechnology, Sungshin Women’s University, Seoul 01133, Republic of Korea; d Microbiology and Nanoscience Lab, Saveetha Medical College and Hospital, Saveetha Institute of Medical and Technical Sciences, Saveetha University, Chennai, 602105, India; e Centre for Biosciences and Biotechnology, Saveetha Institute of Medical and Technical Sciences, Saveetha University, Chennai, 600077, India

**Keywords:** Goji berries, phytochemicals, polysaccharides, carotenoids, functional foods, food processing, nutraceuticals

## Abstract

Goji berries (*Lycium barbarum* and *Lycium chinense*) are widely recognized as functional foods owing to their rich nutritional composition and diverse range of bioactive phytochemicals. This review summarizes the current knowledge on their macro- and micronutrients, polysaccharides, phenolics, carotenoids, vitamins, fatty acids, and other bioactive compounds, highlighting their roles as functional foods and nutraceuticals. These bioactive constituents possess antioxidants and anti-inflammatory activities that may contribute to health promotion and improve dietary quality of life. Recent advances in food processing and applications in bakery, dairy, beverage, snack, and natural preservative formulations are critically evaluated in this review. The effects of cultivation practices, pesticide residues, and post-harvest processing on phytochemical stability, product quality, and safety are also discussed. Future research should prioritize bioavailability, processing stability, clinical validation, and standardization to maximize the nutritional, functional, and commercial potential of goji berry-based products.

## Highlights

• Goji berries are nutrient-rich functional fruits with diverse biological activities.
• Thermal processing significantly alters the stability of carotenoids and phenolics.
• Encapsulation improves stability and delivery of goji berry bioactives.
• Goji by-products show potential for sustainable functional food applications.
• Post-harvest treatments influence phytochemical retention, safety, and product quality.

## Graphical abstract

**Figure:**
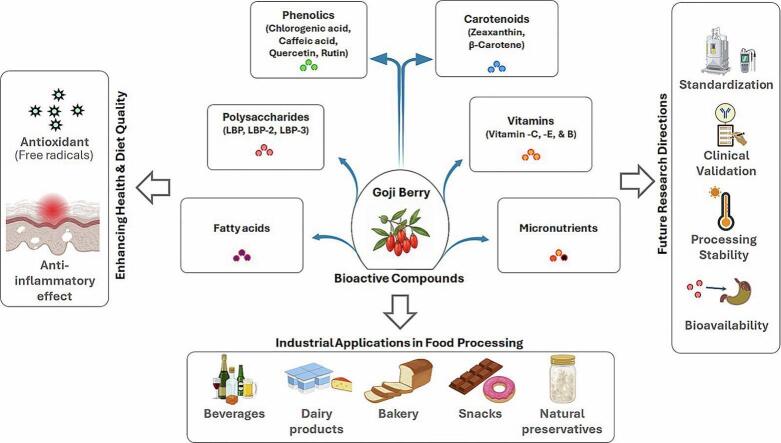

## 1 Introduction

Berry fruits are consumed in various forms, such as fresh, frozen, or dried, and are incorporated into a wide range of food products and dietary supplements. They contain highly valuable bioactive compounds that benefit human health, such as carotenoids and phenolic compounds, which have antioxidant, anti-inflammatory, antidiabetic, anticancer, and other health benefits (Golovinskaia & Wang, 2021). Goji berries are gaining prominence in various fields, including the medical and pharmaceutical industries. Goji berries (*Lycium* spp., Solanaceae family) are derived from the closely related plant species *Lycium chinense* and *Lycium barbarum*, which are distributed across Europe, the United States, and Asia (especially in Japan, China, and Taiwan). The fruits of *L. barbarum* and *L. chinense* (known as goji berry or wolfberry) are yellow to reddish-orange and elongated, whereas the fruits of *L. ruthenicum* (black wolfberry, black goji) are dark purple or black, round, and small (Milinčić et al., 2025; Yao et al., 2021). Over the past 2000 years, goji berries have been extensively used in Chinese medicine because of their nutritional and medicinal properties. Recently, the United Kingdom and North America have increased the consumption of goji berries as dietary supplements and health products (Vidović et al., 2022). They are consumed fresh, dried, or processed and are incorporated into numerous food and beverage products, including infusions, juices, wine, liqueurs, jams, sauces, salads, and beer. Additionally, they serve as functional additives in ice cream, baked goods and dairy formulations (Jurikova et al., 2025). Their medicinal properties are attributed to several bioactive compounds, including carotenoids and phenolic compounds. Although rich in macronutrients, goji berries are a good source of micronutrients, including minerals and vitamins such as riboflavin, thiamine, copper, and manganese (Poggioni et al., 2022). The reported health effects include improved hematopoiesis, anti-aging, anticancer, immune system, and antioxidant activities (Vidović et al., 2022).

The chemical profile and biological activity of goji berries vary across regions owing to differences in genotype, environmental conditions, and pre- and post-harvest practices. In addition, goji berries are widely used as functional ingredients in specialized beverages and various food products, such as baked goods, confectioneries, meat, and dairy products, contributing to their nutritional profile, health-promoting effects, and sensory potential (Jiang et al., 2021). Because of their high bioactive content, the manipulation of these components, particularly those associated with fruit quality parameters, is important for crop improvement. Owing to the presence of polysaccharides and carotenoids, goji berries have immune regulatory and antioxidant effects (Lu et al., 2021), and their demand for goji berries has been increasing worldwide. Goji berries are often available as food or food products in the global functional food market. This review summarizes and critically discusses the bioactive compounds and health benefits of *Lycium* species (*L. barbarum* and *L. ruthenicum*), their molecular mechanisms of action, and their roles in the pharmaceutical and food industries. Moreover, we highlighted food-processing technologies and their influence on the bioactivity of goji berries. We have also included the applications of goji berries, including food and biomedical applications. However, goji berries are highly susceptible to pests and diseases; therefore, pesticide spraying is necessary to harvest high-quality goji berries (Zhi et al., 2024). With respect to safety, studies have shown that despite the effective bioactive potential of goji berries, excessive intake may lead to health-related issues; thus, consumption of a balanced diet outweighing the risks of goji berries would be more appropriate (Berisha et al., 2025). In this review, we discuss the nutritional and phytochemical composition and major bioactive compounds of goji berries, along with their associated health benefits. Furthermore, our review highlights the applications of goji berries in the food industry, the impact of processing technologies on their bioactivity, and their role in the development of functional foods and nutraceutical products in various forms.

## 2 Nutritional and phytochemical composition

Goji berries are considered nutrient-dense fruits rich in both macronutrients and bioactive phytochemicals, and their consumption is increasing due to their associated health benefits. They contain a wide range of compounds, including polysaccharides, organic acids, carotenoids (such as zeaxanthin), vitamins (B, C, and E), flavonoids, phenolic compounds, minerals, trace elements (Zn, Fe, Ca, Se, Ge, P, K, Na, Mg, Co, Mn, S, and B), eight essential amino acids, alkaloids, fats, and other antioxidants with high biological activity (Shi et al., 2024). Goji berry polysaccharides are water-soluble glycoconjugates with molecular weights of 8–214 kDa, representing 5–8% of the fruit’s dry weight, and are the most studied components. Polysaccharides are composed of six types of monosaccharides: arabinose, rhamnose, xylose, mannose, galactose, glucose, and galacturonic acid (Zheng, Wang, et al., 2024). Among the other carotenoids, zeaxanthin dipalmitate was the predominant component, accounting for 55.44% of the total carotenoid content. Key goji berry polyphenols include caffeic acid (3.73 μg/g), chlorogenic acid (12.4 μg/g), *p*-coumaric acid (6.06 μg/g), quercetin, quercetin-diglucoside (66.0 μg/g), isoferulic acid, chlorogenic acid (12.4 μg/g), kaempferol, kaempferol-3-O-rutinoside (11.3 μg/g), cinnamoylquinic acid and its derivatives, and rutin (42.0 μg/g) (Jiang et al., 2021). A human intervention study revealed that after ingestion of 80–100 mg of quercetin from quercetin-rich foods, including apples, onions, or meals rich in plant products, the peak plasma concentrations generally reached only 0.3–0.75 μM, whereas fasting concentrations under habitual dietary conditions were usually <50–80 nM (Cao et al., 2010).

**Figure 1:**
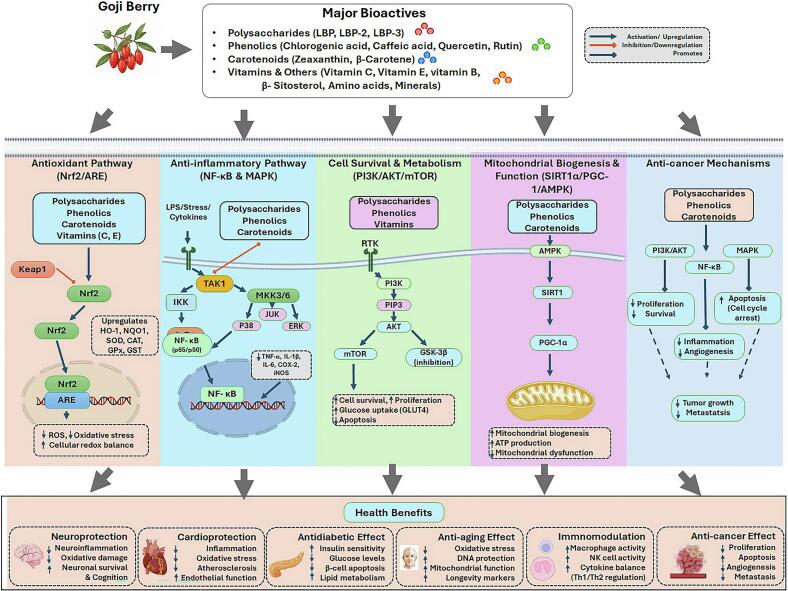
Mechanistic relationships between major goji berry bioactives and their health benefits. Polysaccharides, phenolics, carotenoids, and vitamins modulate antioxidants, anti-inflammatory, immunomodulatory, and metabolic pathways, collectively contributing to neuroprotective, cardioprotective, anti-aging, antidiabetic, and anticancer effects.

Zhu et al. (2024) reported that 100 g of dried goji berries contain approximately 6.6 mg of quercetin-diglucoside (66 μg/g). The concentrations of quercetin commonly employed *in vitro* (5–100 μM) substantially exceed those achievable through normal dietary consumption of goji berries. Therefore, the biological effects observed *in vitro* at supraphysiological concentrations should be interpreted cautiously when extrapolated to dietary intakes. The health benefits of goji berries are more likely attributable to chronic dietary exposure, synergistic interactions among multiple phytochemicals, and the activity of their metabolites**,** rather than the pharmacological effects of individual isolated compounds. Table 1A lists the phytochemicals and their derivatives. Figure 1 illustrates the diverse health benefits associated with goji berries, including their anti-aging, antidiabetic, anticancer, antioxidant, and antimicrobial properties, immune-enhancing effects, and potential roles in regulating metabolism and protecting against age-related and neurological diseases. The nutritional value of goji berries differs among varieties, as shown in Table 1B. The main fatty acids in red, yellow, and black goji berries are myristic, myristoleic, palmitic, palmitoleic, heptadecanoic, stearic, oleic, and linoleic acids, as well as saturated, monounsaturated, and polyunsaturated fatty acids (Ilić et al., 2020). All these compounds exhibit strong antioxidants, anti-inflammatory, and immunomodulatory activities. However, external factors, including geographical origin, cultivation practices, and processing methods, can influence the concentration and bioavailability of these nutrients and phytochemicals in goji berries (Ju et al., 2020). Table 1C shows the bioavailability and experimental levels of goji berries.

**Table 1A:** Phytochemicals and their derivatives are presented in goji berries including preparation methods, doses, and modes of application relevant to biofunctional activities.

**S. No**	**Phytochemicals and its derivatives**	**Sample preparation and analytical conditions**	**Doses / concentration ranges**	**Modes of application and bioactivities**	**References**
1.	**Flavonoids**: Rutin, quercetin, kaempferol, myricetin, hesperidin, anthocyanin, naringin, glulycibarbarspermidine F, petunidin-3-O-rutinoside(glucosyl-p-coumaroyl)-5-O-glucoside, 2-glukukoamine, 3,5-dicaffeoylquinic acid, apigenin, alfalfa, kaempferol, isorhamnosum, pelargonidin	Mostly extracted using aqueous ethanol or methanol (50–80%), ultrasound-assisted or reflux extraction under controlled temperature (25–60 °C)	*In vitro*: 5–100 μM; *in vivo* dietary supplementation equivalent to 50–300 mg/kg body weight	Oral administration or incorporation into functional foods; associated with antioxidants, anti-inflammatory, anticancer, and cardioprotective activities via modulation of NF-κB and MAPK pathways	(Baimakhanova et al., 2025)
2	**Phenolic acids**: Chlorogenic, coumaric, ferulic, protocatechuic, gallic, ellagic, *p*-hydroxycinnamic, citric, isocitric, caffeic and syringic acids	Extracted using polar solvents (water–ethanol mixtures) followed by HPLC-DAD or LC-MS analysis	*In vitro*: 10–200 μg/mL; animal studies: 20–150 mg/kg	Primarily oral intake; contribute to antioxidant, antidiabetic, and hepatoprotective effects by scavenging ROS and regulating glucose-metabolic enzymes	(Marinaccio et al., 2025)
3	**Carotenoids:** Zeaxanthin, zeaxanthin dipalmitate, β-carotene, neoxanthin, cryptoflavin, all-trans-zeaxanthin and all-trans-β-carotene and their isomers, β-cryptoxanthin	Extracted using non-polar solvents (hexane/acetone mixtures), often stabilized by saponification and protected from light and oxygen	Human supplementation studies: 2–15 mg/day (zeaxanthin equivalents)	Dietary intake or encapsulated formulations; associated with eye health, neuroprotection, and anti-aging effects through antioxidant and anti-apoptotic mechanisms	(Baimakhanova et al., 2025)
4	**Alkaloids**: Lyrium spermidine, harmine, atropine, anisodamine, scopolamine, pyrole alkaloid, kukoamine A	Isolated using acid–base extraction followed by chromatographic purification	Low-dose range: μg–mg/kg due to high bioactivity	Oral or experimental pharmacological models; involved in neuroprotective, antihypertensive, and antimicrobial activities, but require safety evaluation due to narrow therapeutic windows	(Zhao & Shi, 2020)
5	**Phytosterol:** Stigmasterol, avenasterol, β-sitosterol, steryl esters, lophenol, dihydrolanosterol, cycloartanol, gramisterol, cycloartenol, citrostadienol, campesta-dienol, cyclobranol	Extracted using lipid-fraction isolation and GC-MS analysis	Dietary intake equivalent: 0.5–3 g/day (total phytosterols)	Consumed as part of whole fruit or enriched food products; linked to cholesterol-lowering, anti-inflammatory, and cardioprotective effects	(Zheng, Schlag, et al., 2024)
6	**Sesquiterpenes**: Vomifoliol, dehydrovomifoliol.	Obtained through solvent extraction followed by GC-MS identification	Reported mainly in qualitative or low-dose experimental studies	Potential signaling molecules with antioxidant and stress-modulating activities; biological roles remain underexplored	(Bendjedou et al., 2021)
7	**Tannins**: Fragariin, sanguiin H-2, ellagitannin	Extracted using aqueous acetone or methanol and characterized by LC-MS/MS	*In vitro*: 10–250 μg/mL	Oral intake associated with antioxidant, antimicrobial, and gut-modulating effects, but excessive intake may reduce mineral bioavailability	(Bernjak & Kristl, 2020)

**Table 1B:** Composition and lipid quality of red, yellow, and black goji berries (Data adopted from Ilić et al., 2020).

S. No	Parameter	Red goji berry	Yellow goji berry	Black goji berry
1.	Moisture (g/100 g)	75.32 ± 0.34	77.52 ± 0.73	84.19 ± 0.36
2.	Ash (g/100 g)	0.84 ± 0.07	0.88 ± 0.09	0.63 ± 0.05
3.	Fat (g/100 g)	1.15 ± 0.08	0.83 ± 0.06	0.71 ± 0.03
4.	Protein (g/100 g)	1.98 ± 0.06	2.24 ± 0.30	1.68 ± 0.01
5.	Total dietary fiber (g/100 g)	3.63 ± 0.25	3.34 ± 0.17	2.76 ± 0.21
6.	PUFA/SFA ratio	2.52	3.59	1.86

**Table 1C:** Translational relevance of major goji berry bioactive compounds.

**Bioactive compounds**	**Actual content in goji berries**	**Bioavailability estimates**	**Dose–dietary achievability assessment**	**Level of evidence**	**References**
LBP	∼5–8% dried fruit	Low	20–60 mg/kg/day achievable;	*In vitro*, animal	(Yang et al., 2022;Wang et al., 2026)
Zeaxanthin dipalmitate	∼ 280 to 540 mg/100 g	High	25mg/kg & 0.5 μm	*In vitro*, animal	(Bahaji Azami & Sun, 2019; Juan et al., 2022)
Lutein	∼ 0.3-1.9 mg/100 g	Moderate	Achievable through diet	Animal, Limited clinical	(Li et al., 2021, Li et al., 2023)
Phenolic compounds	4-13 mg/g DW	Low–moderate	1.89 to 11.36 μg GAE/g; achievable through diet	Mainly *in vitro* & animal	(Jurikova et al., 2025; Rodrigues et al., 2026)
Flavonoids	380 – 1600 μg/g	Moderate	Supplements often needed for therapeutic doses	*In vitro* & animal	(Hou et al., 2025; Liu et al., 2020)
Phenylamides	Abundant	Unknown	40 – 50 μmol/L	Predominantly *in vitro*	(Shunkai et al., 2025; Zhou, Che, et al., 2025)
Caffeoyl-spermidine derivatives	2 to 4.98 mg/g of berry	Unknown	Dietary relevance uncertain	Animal	(Chen et al., 2026; Shen et al., 2026)

## 3 Bioactive compounds and their health benefits

**Figure 2:**
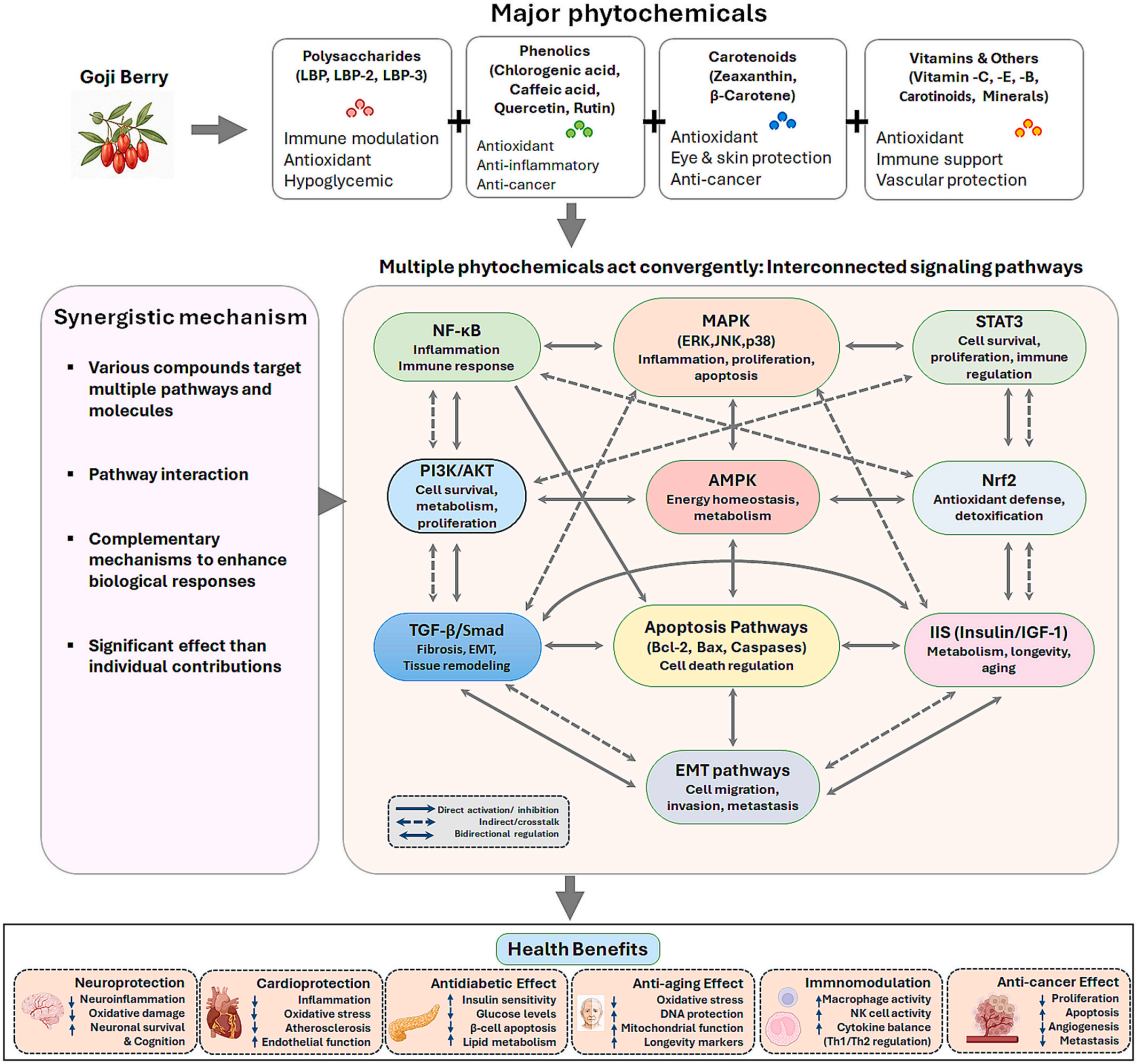
Mechanistic integration of goji berry bioactive compounds and their associated signaling pathways. Major phytochemicals regulate key molecular pathways, including NF-κB, PI3K/AKT, MAPK, TGF-β/Smad, Nrf2, STAT, and insulin/IGF-1 signaling, collectively contributing to anti-inflammatory, antioxidant, neuroprotective, renoprotective, photoprotective, anti-aging, and anticancer effects. The phytochemicals act synergistically to exert antioxidant, antidiabetic, anticancer, anti-aging, anti-inflammatory, and antihypertensive.

Bioactive food compounds are physiologically active constituents derived from plant- and animal-based foods and dietary products that contribute to human nutrition. Goji berries have been considered a “functional food” owing to their antioxidant and nutritive properties, which are beneficial for human health and well-being. The constituents of bioactive foods include carotenoids, flavonoids, isoflavones, phytoestrogens, phytosterols, stanols, vitamins, fibers, fatty acids, probiotics, and prebiotics (Shi et al., 2024). Mocan et al. (2015) reported species-specific differences in the phytochemical composition and antioxidant activity of *L. barbarum* and *L. chinense* leaves. *L. chinense* exhibited significantly higher total phenolic and flavonoid contents, particularly rutin and quercetin derivatives, than *L. barbarum*. *Lycium barbarum* polysaccharides (LBPs) and carotenoids (notably zeaxanthin dipalmitate) are key contributors to the antioxidant and immunomodulatory activities of *L. barbarum.* In contrast, *L. chinense* contains different bioactive constituents such as alkaloids and phenolic compounds (Potterat, 2010). Yossa Nzeuwa et al. (2022) reported that the ethanolic extracts of *L. ruthenicum*, *L. barbarum*, and *L. chinense* exhibited significant antioxidant activities, with *L. ruthenicum* exhibiting the highest radical-scavenging potential. HPLC analysis identified abundant phenolic compounds, including hydroxycinnamic acid amides, chlorogenic acid, and rutin, highlighting the promising application of *Lycium* fruits in the food, nutraceutical, and biomedical fields owing to their strong antioxidant properties (Yossa Nzeuwa et al., 2022). dos Santos et al. (2022) reported a wide diversity of polyamine-derived phytoconstituents in *L. barbarum*, including novel glucosyl-caffeoylspermidine and caffeoylspermine isomers with varying degrees of glycosylation. Using advanced chromatographic and mass spectrometric analyses, this study characterized nearly 100 distinct structures, revealing complex oligosaccharide-linked polyamine conjugates that contribute to the phytochemical diversity of goji berries. The phytochemical composition and antioxidant activity of goji berries are strongly influenced by their geographical origin and environmental conditions (Donno et al., 2015). Zhao and Shi (2022) characterized the phenolic composition and antioxidant activity of *L. barbarum* fruits at different ripening stages (green, orange, and red) using UPLC–MS/MS. The data showed that a wide range of phenolic compounds, including phenolic acids (e.g., chlorogenic and caffeic acids) and flavonoids (e.g., rutin and quercetin derivatives), were present in the four varieties of goji berries. In this section, we highlight the bioactive compounds in goji berries and their associated health benefits (Figure 2), with Table 2 summarizing the characterization techniques used to analyze the composition of goji berries.

**Table 2:** Characterization techniques used for identifying the composition of goji berries including sample preparation, analytical conditions, and quantitative relevance.

**S. No**	**Goji berry**	**Characterization technique**	**Sample preparation and analytical conditions**	**Identified compounds**	**References**
1.	Black, yellow, red goji berries	Ultrahigh-performance liquid chromatography (UHPLC)	Freeze-dried samples extracted with aqueous methanol under controlled temperature; separation using C18 column with gradient elution	Detected: AA-2βG. Not detected: AA-2βG in *L. ruthenicum*. 33.4 and 24.2 mg/100 g for *L. barbarum* (red and yellow) respectively.	(Ilić et al., 2020)
2.	Goji berries samples	UHPLC	Carotenoids extracted using acetone/hexane under light-protected conditions; analysis performed at low temperature to prevent degradation	High contents of carotenoid compounds	(Ayuso-Yuste et al., 2024)
3	Goji berries from different regions including Gansu, Ningxia, Qinghai, and Xinjiang of China and Mongolia	UHPLC-MS, HPLC-ELSD	Aqueous extraction followed by filtration and dual detection to cover both polar and non-chromophoric compounds	Amino acids (20), nucleosides (17) and nucleobases, sugars (4) and proteins were detected	(Lu et al., 2021)
4	First harvest stage of goji berry fruits	Targeted metabolome and transcriptome. analysis	Metabolites profiled using LC-MS coupled with gene expression analysis under defined developmental stages	Chlorogenic acid and its positional isomers (neochlorogenic acid and cryptochlorogenic acid) increased significantly along with P-coumaroyl quinic acid as the harvest stages progressed, while the other bioactive DEMs including scopoletin, scopolin, naringenin, and pruning exhibited a decreasing trend	(Cui et al., 2024)
5	Fresh/dry goji berries during ripening stages	HPLC	Aqueous extraction with filtration; analysis under controlled pH conditions	Sugars (sucrose, fructose, and glucose) and organic acids (mallic, citric, and succunic acid)	(Oğuz et al., 2021)
6	Goji berries at different developmental stages	Gas chromatography/mass spectrometry	Derivatization of volatile and semi-volatile compounds prior to GC-MS analysis	Fatty acids, alkanes, aromatic acids, and low-molecular-weight organic acids	(Zhou, chen, et al., 2025)

### 3.1 Goji berry polysaccharides and their biological benefits

Polysaccharides constitute approximately 5–8% of the dry weight of goji berries and include pectic polysaccharides, glucans, xylans, and arabinogalactan proteins. These water-soluble polysaccharides are the most important functional components of goji berries. The glycan backbone of *L. barbarum* polysaccharides comprises (1→3)-*β*-D, (1→6)-*β*-D, and (1→4)-*α*-D-galactopyranosyl uronic acid residues (Vidović et al., 2022). A critical consideration when interpreting the biological effects of LBPs is their limited oral bioavailability. LBPs are classified as non-starch polysaccharides (NSPs) that resist digestion by human gastrointestinal enzymes and are therefore not efficiently absorbed across the intestinal epithelium. Instead, they reach the colon largely intact, where they undergo microbial fermentation to generate bioactive metabolites, including short-chain fatty acids (SCFAs), which are believed to mediate several systemic effects (Lai et al., 2022). Consequently, the therapeutic benefits of LBPs should be interpreted primarily through microbiota-mediated, immunomodulatory, and metabolic mechanisms, rather than the direct absorption of intact polysaccharides. Recently, oral administration of LBP capsules to patients with nonalcoholic fatty liver disease (NAFLD) enhanced GGT, ALB, TP, and D-BIL levels (Fan et al., 2025). Although these findings provide preliminary clinical evidence, the underlying mechanisms remain uncertain because intact LBPs are poorly absorbed, suggesting that microbiota-derived metabolites may contribute to the observed therapeutic response.

In a rat model of Alzheimer’s disease (AD), oral administration of LBPs improved cognitive performance, reduced Aβ pathology, decreased oxidative stress and neuroinflammation, and restored cholinergic neurons, thereby exerting neuroprotective effects (Lu et al., 2025). Considering their limited systemic bioavailability, these neuroprotective effects are more likely mediated through modulation of the gut–brain axis and systemic immune regulation than through direct penetration of LBPs into neural tissues. Comparative studies between *L. barbarum* water extracts and polysaccharides revealed that both enhance bone mass and strength by directly binding to BMP inhibitors (noggin), which phosphorylate downstream signaling molecules that ultimately improve bone loss and stimulate bone formation (Sun, He, et al., 2025). However, confirming these mechanisms in humans requires pharmacokinetic studies to establish whether sufficient concentrations of bioactive molecules or their metabolites reach skeletal tissues after oral administration. Nanoparticles synthesized with LBPs (LBP-SeNPs@TP) upregulated Bax, Cyt C, and caspases while downregulating Bcl-2, thereby inhibiting the growth of pancreatic tumor cells both *in vitro* and *in vivo* (Li, Su, et al., 2025). Importantly, nanoparticle-based formulations may overcome the intrinsic bioavailability limitations of native LBPs by enhancing their stability, cellular uptake, and targeted delivery, thereby improving their translational potential. LBPs also treat ulcerative colitis by strengthening gut barrier function and remodeling the gut microbiota, increasing beneficial bacteria (*Muribaculaceae)* and reducing harmful bacteria (*Bacteroides)* (Liu et al., 2025). Among the reported therapeutic applications, ulcerative colitis provides the strongest mechanistic support because the primary site of LBP activity is the gastrointestinal tract, where direct interaction with the gut microbiota is expected despite poor intestinal absorption. In cadmium (Cd)-induced testicular damage, LBPs decreased testicular hormonal disorders and oxidative stress damage and regulated iron and cellular energy metabolism (Zhang, Zhang, et al., 2025). LBPs also improve male fertility by inhibiting oxidative stress and downregulating ZnT3 signaling, ultimately improving oligoasthenozoospermia in mice (Li, Su, et al., 2025). Nevertheless, these findings are predominantly derived from animal models, and their clinical relevance remains uncertain in the absence of human pharmacokinetic and efficacy studies.

Despite extensive pharmacological evidence supporting LBPs, most mechanistic studies have been conducted *in vitro* and *in vivo*, whereas clinical evidence remains limited. However, the clinical translation of these findings remains constrained by the poor oral bioavailability of intact LBPs, species-specific differences in metabolism and pharmacokinetics, and incomplete understanding of microbiota-mediated mechanisms (Gao et al., 2025). Furthermore, current extraction technologies often produce low yields and exhibit variability in polysaccharide composition, posing challenges for standardization and large-scale pharmaceutical development. Thus, high-quality clinical studies and the development of innovative delivery platforms, including nanoparticles, liposomes, nanoformulations, and targeted conjugates, to improve the stability, bioavailability, and therapeutic efficacy of LBPs are essential (Wang et al., 2020). Amyloid fibrils and LBPs have been developed as drug delivery systems for curcumin. *In vitro* digestion of LBP-amyloid fibrils enhanced the bioaccessibility of curcumin by 28.96% compared to curcumin in oil (Li, Kang, et al., 2025). These findings further highlight that engineering delivery systems is a rational strategy to overcome the poor bioavailability of native LBPs while simultaneously enhancing the delivery of co-encapsulated therapeutic compounds. LBPs exhibit protective effects against ischemia-reperfusion injury (IRI) in hippocampal (HT22) cells by decreasing LDH release, reactive oxygen species (ROS) levels, and apoptosis via the SIRT1/PGC-1α pathway (Niu et al., 2025). A hydrogel injectable system incorporating LBPs, sodium alginate, and strontium chloride (SrCl_2_) demonstrated rapid cross-linking and gel formation, as well as sustained release of active ingredients in the bone defect region, thus promoting the regeneration of bone and vasculature (Xie et al., 2025). Zein- and LBP-encapsulated chlorogenic acid nanoparticles exhibit enhanced bioaccessibility, stability, antioxidant potential, and cellular protection (Ma, Guo, et al., 2025). Collectively, these formulation-based studies suggest that the future therapeutic potential of LBPs may rely more on advanced delivery technologies than on the administration of native polysaccharides alone.

Cao et al. (2022) extensively reviewed that LBP acts as a prebiotic by efficiently enhancing beneficial bacteria (e.g., *Bifidobacterium* and *Lactobacillus*), increasing SCFAs production, and exhibiting immunomodulatory effects by activating immune cells and regulating cytokines, thereby improving gut health, immunity, and metabolic regulation. This prebiotic activity provides a plausible biological explanation for the systemic effects reported in experimental studies, despite the limited absorption of intact LBPs. LBPs reduce inflammation by attenuating IL-18, TNF-α, and IL-1β levels; reducing oxidative stress (by reducing ROS levels and improving SOD and total antioxidant activity); attenuating apoptosis; and regulating mitochondrial acetylation homeostasis by activating Sirt3 signaling. These effects reduce intracerebral hemorrhage, highlighting the promising potential of this approach for future clinical applications. Geng et al. (2025) reported that LBPs attenuate rheumatoid arthritis by restoring the balance between CCR9^+^ Th17 and Treg cells in the peripheral blood and synovial fluid of patients with rheumatoid arthritis. Overall, while LBPs exhibit diverse pharmacological activities across multiple disease models, the available evidence should be interpreted in light of their limited oral bioavailability. Future studies should integrate pharmacokinetic analyses, microbiome profiling, and well-designed randomized clinical trials to determine whether the observed therapeutic benefits are mediated by intact polysaccharides, microbial metabolites, or secondary host responses, thereby facilitating rational clinical translation of LBP-based therapeutics.

#### 3.1.1 Gut microbiota mediation

The polysaccharide chains of LBPs are primarily digested and utilized by gut microbes instead of the host and are poorly digested by human gastrointestinal enzymes; therefore, they are minimally absorbed in the small intestine. Additionally, LBPs are resistant to digestion under simulated oral, gastric, and intestinal conditions, allowing them to reach the colon in a largely intact state. *In vitro* fermentation studies have demonstrated that these polysaccharides are readily utilized by the gut microbiota, as evidenced by the reduction in total carbohydrate content and the increased production of SCFAs, including acetate, propionate, and butyrate (Ding et al., 2019). Furthermore, LBP fermentation significantly enriched the beneficial bacterial taxa, including members of the phyla Bacteroidetes (*Bacteroides* and *Prevotella*), Firmicutes (*Lactococcus* and *Faecalibacterium*), and Actinobacteria (*Bifidobacterium*). The ability of these microorganisms to degrade LBPs is likely mediated by specialized carbohydrate-active systems, such as starch utilization system (Sus)-like complexes in Bacteroidetes and ATP-binding cassette (ABC) transporter-associated carbohydrate utilization pathways in Firmicutes and Actinobacteria, suggesting that LBP degradation occurs through the cooperative metabolic activity of the gut microbiota (Cao et al., 2022; Koropatkin et al., 2012).

SCFAs are absorbed by colonocytes via monocarboxylate transporters (MCT1) and sodium-coupled monocarboxylate transporter-1 (SMCT1) before entering the portal circulation (Parada Venegas et al., 2019). Following absorption via monocarboxylate transporters (MCT1 and SMCT1), SCFAs influence cell proliferation, differentiation, and gene expression and activate G protein-coupled receptors (GPR41, GPR43, and GPR109A), leading to anti-inflammatory signaling (Thorburn et al., 2014). These metabolites subsequently reach the liver and systemic circulation, where they regulate glucose and lipid metabolism, immune responses, and intestinal barrier integrity (Silva et al., 2020). SCFAs reach the colon intact, where they undergo fermentation by gut microbiota, in a reported outcome as neuroprotective, it was found that LBPs can modulate maternal gut microbiota, improve the intrauterine environment of the mother and reduce the stress factors on the emotional damage of offspring (Zhao et al., 2021). Thus, the *in vivo* effects of LBPs are likely mediated indirectly through gut microbiota metabolism or local intestinal immune modulation. In a report, it was suggested that the apparent permeability coefficient (P_app_) LBP was less than 1.0 × 10 ^−6^, and thus it is hardly absorbed in the intestine and demonstrates that direct systemic distribution is minimal (Wang et al., 2021). The same study reported that the immune response induced by LBP was associated with the regulation of gut microbiota. LBP is not degraded in the upper digestive system and reaches the large intestine, where it is fermented by the colon microbiota to produce beneficial metabolites such as SCFAs (Deng et al., 2018). Several studies have demonstrated that LBPs exert biological effects by modulating the gut microbiota and acting as prebiotic substrates that are metabolized by intestinal microbes (Cao et al., 2022). Only a limited number of investigations have employed stronger mechanistic approaches, such as antibiotic depletion models, germ-free animals, and fecal microbiota transplantation (FMT), which are necessary to establish a direct causal relationship between microbiota changes and host health outcomes (Huang et al., 2023). Consequently, it remains unclear whether specific bacterial taxa directly mediate the therapeutic effects of LBPs. Generally, LBPs have demonstrated prebiotic potential by restoring gut microbial composition and enhancing SCFA production in an *in vitro* fermentation model of Wilson disease. Compared with penicillamine, LBPs more effectively enriched beneficial SCFA-producing bacteria and increased acetate and total SCFA levels, suggesting their potential as microbiota-targeted adjunctive therapies for rheumatoid arthritis. However, these findings require validation through *in vivo* and clinical studies (Fang et al., 2026).

### 3.2 Phenolic compounds in goji berries and their biological benefits

#### 3.2.1 Flavonoids

The flavonoids include myricetin, mulberin, rutin, quercetin, kaempferol, quercetin-rhamnose-dihexoside, quercetin-3-*O*-rubutin, naringin, aurantiamarin, neohesperidin, naringenin, hesperetin, and kaempferol-3-*O*-rubutin (Qiang et al., 2023). Tables 3A and B summarize the major flavonoids with beneficial effects on human health. Unlike polysaccharides, flavonoids are partially absorbed in the small intestine; however, their oral bioavailability varies considerably because of their poor aqueous solubility, extensive phase I/II metabolism, rapid elimination, and transformation by gut microbiota. Consequently, the circulating concentrations of native flavonoids after dietary intake are generally low, and many of their biological effects are mediated by metabolites rather than by the parent compounds. Therefore, the pharmacological evidence discussed below should be interpreted in the context of achievable physiological exposure rather than the pharmacological doses commonly used in experimental studies. Niu et al. (2022) reported that *L. barbarum* leaf flavonoid extracts upregulated erythropoietin and *heme oxygenase-1* (*HO-1*) genes by targeting the MAPK signaling pathway in human umbilical vein endothelial cells (HUVEC). Furthermore, they extended the lifespan of *Caenorhabditis elegans* by enhancing mobility in mid-life stages and increasing *sod-2*, *gcs-1,* and *skn-1* expression, highlighting the anti-aging effects of the extract (Niu et al., 2022). However, these findings originate from cell culture and invertebrate models, and whether similar molecular responses occur in humans at dietary intake levels remains unclear. Quercetin significantly inhibited the release of inflammatory mediators, such as TNF-α, MCP-1, and IL-1β, and decreased the levels of ICAM-1 and VCAMs in LPS-stimulated HUVECs, indicating that *Lycium* species contain high levels of antioxidant components with strong nutritional value (Lan et al., 2024). Although quercetin possesses well-established anti-inflammatory properties, its low oral bioavailability suggests that the observed cellular effects may not directly translate into systemic efficacy following normal dietary consumption of goji berries. Myricetin (100 mg/kg) decreased formaldehyde-exacerbated asthmatic inflammation by chelating Fe(II) in pulmonary macrophages, highlighting its potential as an anti-inflammatory agent (Ma, Wang, et al., 2025). Notably, the dose employed substantially exceeded the amount achievable through normal dietary intake, emphasizing the need to distinguish pharmacological doses from nutritional exposures. Naringin administered for two weeks downregulated caspase-3, IL-17, and NF-κB expression in cerebellar tissue, thereby suppressing IRI-induced inflammation in rats (Babacanoglu et al., 2025). Kaempferol flavonoids from roasted *L. chinense* leaves demonstrated anti-obesity effects by inhibiting pancreatic lipase activity and reducing lipid accumulation (Choi et al., 2019). Hesperidin exerts dose-dependent hepatoprotective effects by reducing the activities of aspartate aminotransferase, alanine aminotransferase, and collagen I and III while enhancing antioxidant activity in deltamethrin-induced hepatotoxicity (Karabekir et al., 2025). In mice with type II diabetes mellitus, hesperetin–Cu (II) complexes modulated the gut microbiota by increasing the levels of beneficial bacteria, decreasing harmful bacteria, maintaining intestinal integrity, and decreasing local inflammation, thereby alleviating diabetes (Peng, Wei, et al., 2025). Hesperetin exerts dose-dependent cytotoxic effects in oral carcinoma KB cells by increasing mitochondrial membrane potential, apoptosis, and oxidative DNA damage, while reducing cell viability (Khair et al., 2025). Collectively, these studies demonstrate the broad pharmacological potential of flavonoids in various diseases. However, most of these studies were conducted using purified flavonoids at pharmacological concentrations or specialized formulations rather than whole goji berry consumption, limiting their direct clinical applicability.

**Table 3A:** Phenolic constituents present in goji berries and their biomedical applications.

**S. No**	**Polyphenolic compounds**	**Health benefits**	**Mechanism**	**References**
	**Flavonoids**			
1.	Rutin and myricetin	Anti-aging effect and stronger antioxidant capacity	Downregulating Aβ expression	(Liu et al., 2020)
2	Anthocyanin extract of *Lycium ruthenicum*	Enhancement of intestinal barrier function	Decreases the synthesis of pro-inflammatory molecules, boosts antioxidant activity, and improves both intestinal morphology and the composition of gut microbiota.	(Wang et al., 2025)
3	Anthocyanin extract of *Lycium ruthenicum*	Anti-aging effect	JNK-1 and DAF-16/FOXO	(Xu et al., 2025)
4.	Anthocyanins from *Lycium ruthenicum* Murray	Protective effect against Parkinson's disease	Modulates anti-inflammatory flora in the gut and endogenous metabolites	(Cao et al., 2024)
5	Quercetin	Anti-inflammatory potential	Inhibits the production of inflammatory factors such as TNF-α, MCP-1, interleukin-1β, ICAM-1, and VCAM-1	(Lan et al., 2024)
6	Hesperidin	Neuroprotective effects	Antioxidant, anti-inflammatory, and anti-apoptotic properties	(Sim et al., 2022)
7	*Lycium barbarum* leaf flavonoid extracts	Anti-aging effects	Upregulates the expression of the *sod-2*, *gcs-1*, and *skn-1* genes	(Niu et al., 2022)
8	kaempferol-3-sophoroside-7-glucoside	Anti-obesity	Its metabolites possess antiobesity activity	(Choi et al., 2019)
	**Phenolic acids**			
9	Ferulic acid	Antioxidant potential	Scavenges free radicals	(Mamdouh et al., 2021)
10	Chlorogenic acid	Prevention of metabolic disease	Antioxidant potential	(Antonelli & Donelli, 2024)
11	Chlorogenic acid	Improves intestinal barrier function	NF-κB signaling pathway	(Yu et al., 2021)

The dose–response relationship of flavonoids remains a major limitation, as their antioxidant and disease-preventive effects are often extrapolated from high-dose experimental studies without accounting for the relatively low systemic concentrations achieved through dietary intake. van den Driessche et al. (2019) conducted a randomized, double-blind crossover trial in healthy overweight men and reported that a single 25 g dose of dried *L. barbarum*, compared with a control meal, did not significantly alter postprandial metabolic parameters in healthy overweight men. In contrast, numerous animal studies have demonstrated the beneficial metabolic, antioxidant, and anti-inflammatory effects of repeated or long-term administration of curcumin. Rather than representing true contradictions, these findings may reflect differences in the time course of bioactivity. Acute consumption of goji berries does not produce measurable effects on postprandial metabolism in humans, such as no significant changes in energy expenditure, substrate oxidation, or key metabolic parameters, such as blood glucose, triglyceride, and free fatty acid levels. These findings highlight an important distinction between acute nutritional intake and chronic biological adaptation, suggesting that beneficial effects may require sustained consumption rather than a single dose of the supplement. Although flavonoids are absorbed more readily than polysaccharides, their metabolites are also substantially influenced by gut microbial biotransformation, supporting the concept that repeated dietary exposure, rather than acute supplementation, is more likely to produce measurable physiological benefits.

Emerging evidence indicates that the consumption of goji berries at doses equivalent to typical human intake induces the pro-oxidant behavior of phenolic compounds, and the same study suggested that goji berry juice may stimulate early redox imbalance and induce hepatic and renal biochemical alterations in female Wistar rats (Rodrigues et al., 2026). These observations suggest that flavonoids may exhibit concentration-dependent biphasic (hormetic) effects**,** rather than acting exclusively as antioxidants. Dietary polyphenols can transiently induce oxidative stress, which may activate endogenous antioxidant defense mechanisms. Furthermore, the findings were obtained in female Wistar rats following short-term exposure, and species-specific differences in metabolism and bioavailability may limit the direct translation of these findings to humans. These findings should also be interpreted in the context of the apparently conflicting evidence from the human intervention studies. Acute human studies primarily evaluate immediate physiological responses, whereas many bioactives in goji berries require repeated consumption to accumulate active metabolites, modulate gut microbiota composition, improve intestinal barrier function, and activate downstream metabolic and immune signaling pathways. Consequently, the absence of measurable effects following a single dose does not necessarily preclude beneficial outcomes associated with chronic dietary intake. Similarly, the pro-oxidant effects observed by Rodrigues et al. (2026) should not be interpreted as evidence of its toxicity. Many dietary polyphenols exhibit hormetic behavior, whereby transient increases in oxidative stress activate endogenous cytoprotective pathways, including Nrf2-mediated antioxidant responses, which ultimately enhance the cellular resilience. Whether the early redox imbalance observed in female Wistar rats represents an adaptive physiological response or the onset of adverse toxicity remains unclear and requires further investigation to clarify this issue**.** Jurikova et al. (2025) reported that although polyphenols are recognized as important bioactive constituents of goji berries, their bioaccessibility and bioavailability remain largely unexplored. In contrast, existing research has primarily focused on the bioaccessibility of carotenoids in *L. barbarum* using simulated gastrointestinal digestion models coupled with metabolomic analyses (Hu, Ma, et al., 2022). Overall, current evidence indicates that flavonoids contribute substantially to the biological activity of goji berries; however, their clinical translation is constrained by variable oral bioavailability, extensive metabolism, uncertain dose–response relationships, and limited human intervention studies. Future research should integrate pharmacokinetic profiling, metabolomics, and randomized clinical trials to identify the bioactive metabolites responsible for the observed health benefits and establish physiologically relevant therapeutic doses.

#### 3.2.2 Phenolic acids

The most common phenolic acids in *Lycium* species include gallic, vanillic, chlorogenic, *p*-coumaric, ferulic, veratric, benzoic, salicylic, syringic, and protocatechuic acids (Jiang et al., 2021). Table 3 summarizes the health benefits of these compounds. Phenolic acids generally exhibit higher intestinal absorption than high-molecular-weight polysaccharides because of their relatively low molecular weights. However, their systemic bioavailability remains highly variable owing to rapid intestinal absorption, extensive phase II metabolism (glucuronidation, sulfation, and methylation), and the elimination of these compounds. Consequently, the circulating concentrations of free phenolic acids following dietary consumption are often substantially lower than those used in experimental studies. Therefore, the biological activities discussed below should be interpreted within the context of their pharmacokinetic limitations and physiologically achievable exposure. Gallic acid (50 mg/kg body weight/day) effectively alters blood urea nitrogen, creatinine, aspartate aminotransferase, alanine aminotransferase, cholesterol, triglyceride, antioxidant enzyme, and malondialdehyde levels, thereby protecting against fenitrothion-induced hepatotoxicity and nephrotoxicity (Apaydin et al., 2025). Although these findings demonstrate promising antioxidant and organ-protective activities, the administered dose was considerably higher than that achievable through the normal dietary intake of goji berries, limiting direct clinical extrapolation. It also exhibits bactericidal potential, improves the efficacy of moxifloxacin and levofloxacin against multidrug-resistant *M. tuberculosis*, and modulates the expression of efflux pump genes (*Rv1410c* and *Rv1258c*), supporting its antibacterial and antiseptic potential (Wei, Bostani, et al., 2025). These observations further suggest that phenolic acids may be valuable adjunctive therapeutic agents rather than stand-alone antimicrobial compounds. Mutlu Sonat et al. (2025) formulated vanillic acid into *in situ* gels for sustained release, improving wound healing without cytotoxicity by decreasing prostaglandin E2 levels and moderately increasing IL-6. The development of sustained-release formulations addresses one of the major limitations of phenolic acids by prolonging local drug exposure and improving their therapeutic efficacy. Ferulic acid decreases peroxisomal oxidative stress by reducing ACOX1 activity, ROS accumulation, and inflammation, while modulating muscle protein metabolism, and is highly promising as a natural targeted therapy for sarcopenia (He et al., 2025). Veratric acid reduces triglyceride and cholesterol levels by downregulating the mRNA expression of key genes involved in fatty acid synthesis, including *SREBP1c*, *FAS*, and *acetyl-CoA carboxylase*, through modulation of the peroxisome proliferator-activated receptor (PPAR) signaling pathway (Pan et al., 2024). Collectively, these studies demonstrate the broad pharmacological potential; however, most evidence has been generated using purified compounds in experimental models rather than through dietary consumption of goji berries. Overall, although phenolic acids exhibit favorable pharmacological properties compared with polysaccharides, their clinical translation remains limited by rapid metabolism, unclear dose–response relationships, and the lack of well-designed human intervention studies.

#### 3.2.3 Carotenoids, coumarins and phenylamides

Carotenoids are the major bioactive pigments in *L. barbarum* (goji berries), contributing to their characteristic red–orange coloration (Zhao, Xu, et al., 2023). Among carotenoids, zeaxanthin is the most abundant, followed by monohydroxy- and dihydroxy-lutein and α-and β-carotene in goji berries (Qiang et al., 2023). Among the major bioactive constituents of goji berries, carotenoids exhibit comparatively favorable bioaccessibility and intestinal absorption. Simulated gastrointestinal digestion coupled with Caco-2 cell transport studies demonstrated bioavailability ranging from 40% to 98%, with the highest intestinal uptake of zeaxanthin dipalmitate (Juan et al., 2022). Consequently, carotenoids possess greater translational potential than many other phytochemicals, whose biological activities are constrained by poor oral bioavailability. In particular, zeaxanthin dipalmitate, an esterified carotenoid isolated from goji berries, has been reported to inhibit blue-light-induced photooxidation of A2E, a toxic retinal byproduct, showing stronger protective activity than vitamin E, prolonged retinal protection by filtering blue light, and scavenging of ROS in early age-related macular degeneration (Basyal et al., 2026). The relatively high bioavailability of zeaxanthin dipalmitate supports the biological plausibility of these retinal protective effects; however, confirmation in long-term human clinical trials is required. Transgenic plants overexpressing the anthocyanin-regulating transcription factor *LrAN2* accumulated significantly higher levels of anthocyanins, which improved their antioxidant defense system by increasing ROS scavenging capacity and reducing oxidative damage under Cd stress (Ai et al., 2025). In HepG2 cells, pretreatment with zeaxanthin dipalmitate significantly enhanced protection against beauvericin (BEA)-induced cytotoxicity, with cytoprotective effects increasing after 48 h of exposure and at higher BEA concentrations (2.5 μM) compared to 24 h and 1.25 μM (Juan et al., 2022). These findings further support the functional relevance of its relatively high intestinal bioavailability and cellular uptake.

Coumarins are important secondary metabolites with anticoagulants, anticancer, antibacterial, and antifungal activities. The most common coumarins in *Lycium* species are scopoletin, fabiatrin, scopolin, and esculin (Rodrigues et al., 2026). In contrast, coumarins exhibit poor oral bioavailability. Pharmacokinetic studies have demonstrated the oral bioavailability of scopoletin at doses of 10, 25, and 50 mg/kg to be 7.08%, 5.87%, and 5.69%, respectively, values comparable to that of coumarin (3.40 ± 2.60%) (Zhao et al., 2019). Therefore, the interpretation of their pharmacological activities should consider the limited systemic exposure achieved after oral administration. Scopoletin isolated from *L. barbarum* exhibited anti-inflammatory effects by decreasing pro-inflammatory cytokines (TNF-α, IL-1β, IL-6) and chemokines (IL-8, CCL17, CCL22) in a 2,4-dinitrochlorobenzene induced animal model (skin disease model) via inhibition of NF-κB, MAPK, and STAT1 signaling pathways (Bak et al., 2022). Although these anti-inflammatory effects are promising, improving oral delivery remains a prerequisite for the successful clinical translation of these drugs. The poor oral bioavailability of scopoletin has prompted the development of numerous structural derivatives and novel pharmaceutical formulations to enhance its therapeutic efficacy. For example, Shi et al. (2017) reported that isoxazole-based hybrids of scopoletin represent an effective chemical modification that significantly improves the anticancer activity of scopoletin. These formulation strategies directly address the pharmacokinetic limitations of scopoletin and represent a promising direction for future drug development.

Phenylamides isolated from goji berries are bioactive compounds with various health-promoting properties. These molecules are conjugates of hydroxycinnamic acids (such as caffeic and ferulic acids) with polyamines such as spermidine and spermine, which stimulate strong antioxidant activity by scavenging ROS, inhibiting lipid peroxidation, and protecting cellular macromolecules from oxidative damage (Mocan et al., 2015). Compared to carotenoids and coumarins, knowledge of the bioavailability and metabolism of phenylamides is limited. Consequently, the interpretation of their biological activities should be made cautiously because pharmacokinetic evidence is insufficient to establish whether the compounds reach the target tissues at biologically relevant concentrations. A Comparative study on polyphenol quantification among *L. ruthenicum, L. barbarum*, and *L. chinense* revealed that hydroxycinnamic acid amide (HCAAs) class derivatives, including N1, N10-bis(dihydrocaffeoyl)spermidine, N1- bishydrocaffeoyl, N10-caffeoyl spermidine, and N1, N10 -di(caffeoyl) spermidine, were dominant in *L. ruthenicum* (15.56-310.80 mg/100 g). Chlorogenic acid was detected in (*L. ruthenicum* (238.59 mg/100 g), *L. barbarum* (25.76 mg/100 g), and *L. chinense* (98.86 mg/100 g). Rutin was found to be the most abundant in *L. chinense*. (62.56±0.061 mg/100 g) (Nzeuwa et al., 2022). High-performance liquid chromatography fingerprinting combined with quadrupole time-of-flight mass spectrometry and chemometric analysis of the origin and phenolic content of *L. ruthenicum*. Among the 26 phytochemicals identified in *L. ruthenicum*, 16 were newly reported, including anthocyanins, flavonoid glycosides, and hydroxycinnamic acid derivatives. Notably, kukoamine A was identified as the predominant compound, highlighting the significance of phenolic amides in *L. ruthenicum* (Yossa Nzeuwa et al., 2017). Metabolomic analysis revealed that black and white fruits contain phenylamides, such as N-caffeoyl-N′-dihydrocaffeoyl spermidine, N,N′-bis(dihydrocaffeoyl) spermidine, and hydroxycinnamic acid amides. In particular, N-caffeoyl-N′-dihydrocaffeoyl spermidine was identified as the predominant constituent of black goji berries (Milinčić et al., 2025). Glycosylated/non-glycosylated putrescine derivatives and caffeoyl-dihydrocaffeoyl-feruloyl spermidines were identified for the first time (Milinčić et al., 2024). LC/DAD-ESI-MS analysis revealed the presence of glu-lycibarbarspermidine F, 2-glu-kukoamine, rutin, and 3, 5-dicaffeoylquinic acid in goji berries. Additionally, an intestinal co-culture model showed that glu-lycibarbarspermidine F (isomer 2) (73.70%), 3,5-dicaffeoylquinic acid (52.66%), and isorhamnetin-3-O-rutinoside (49.31%) traversed the intestinal cell layer and exerted beneficial health-promoting effects (Teixeira et al., 2024). These findings provide preliminary evidence that selected phenylamides possess intestinal permeability; however, *in vitro* transport studies alone cannot predict systemic bioavailability or therapeutic efficacy in humans. Overall, despite the increasing interest in goji berry phenylamides, the current evidence remains largely descriptive and dominated by phytochemical characterization and *in vitro* studies. Comprehensive pharmacokinetic investigations, metabolomic analyses, and well-designed clinical studies are required to clarify the metabolism, bioavailability, and contribution of these compounds to the health benefits of goji berries (Wei, Wang, et al., 2025).

#### 3.2.4 Other active compounds

Compared with polysaccharides and carotenoids, many of the minor phytochemicals identified in *L. barbarum* occur at relatively low concentrations, and information regarding their oral bioavailability, metabolism, and tissue distribution is limited. Consequently, although these compounds exhibit diverse biological activities in phytochemical and experimental studies, their direct contribution to the health benefits of goji berries in humans remain uncertain. Their physiological effects may depend on biotransformation, synergistic interactions with other phytochemicals, or metabolism by the gut microbiota, rather than high systemic absorption. Therefore, evidence describing these compounds should be interpreted cautiously, particularly when extrapolating *in vitro* findings to clinical settings.

The fruits and roots of *L. barbarum* contain a diverse array of minor bioactive constituents, including lignans (cannabisin, cannabisin E and F, threocanabisine, erythrocanabisine H, melongenamide D, and grossamide), phenylpropanoids and their derivatives, terpenoids, alkaloids (lycibarbar spermidine T, lycibarbar spermidine C, and lycibarbarspermidine A), sterols, steroids, anthraquinones/anthraquinone glycosides, glycerogalactolipids, dihydroferulic acid, ethyl dihydroferulate, N-trans-feruloyl tyramine, digupilgan A, and p-hydroxybenzaldehyde (Jiang et al., 2024; Ma et al., 2023). Although many of these compounds possess antioxidant, anti-inflammatory, or cytoprotective properties in experimental models, their concentrations in edible fruits are generally low, and evidence demonstrating sufficient exposure following dietary consumption remains limited. Future studies should prioritize pharmacokinetic characterization and clinical validation to determine which of these minor constituents meaningfully contribute to the biological activity of goji berries.

### 3.3 Fatty acids

Despite their recognized nutritional importance, fatty acids account for only approximately 1–4% of the dry weight of goji berries. Consequently, the physiological contributions of goji-derived fatty acids should be interpreted in the context of their relatively low abundance and dietary intake. Although unsaturated fatty acids, such as linoleic and α-linolenic acids, possess established cardiovascular and neurological benefits, the quantities obtained from typical goji berry consumption are unlikely to represent a major nutritional source compared with conventional edible oils, nuts, or seeds. Therefore, their biological relevance may be greater in relation to fruit quality, aroma development, and potential synergistic interactions with other phytochemicals than as primary bioactive compounds. Fatty acids in *Lycium* species are classified into saturated, monounsaturated (MUFA), diunsaturated, and polyunsaturated (PUFA) fatty acids, along with two long-chain amides (Moustafa et al., 2025). Approximately 22 fatty acids have been identified in dried goji berries, including oleic, linolenic, linoleic, and palmitic acids (Zheng, Oellig, Vetter, et al., 2025). Linoleic acid is the predominant fatty acid, followed by smaller amounts of α-linolenic, oleic, palmitic, and stearic acids, which have been extensively associated with cardiovascular and neurological health (Vidović et al., 2022). However, these health associations are largely derived from studies investigating purified fatty acids or other dietary sources rather than goji berries specifically, and direct clinical evidence attributing these benefits to goji berry fatty acids is limited.

Unsaturated fatty acids play a crucial role in aroma development. For instance, linoleic acid is a precursor of aromatic constituents such as hexanal, nonenal, and octen-3-one. Few studies have assessed the fatty acid composition of fresh goji berries. Furan fatty acids (FuFAs) are present in multiple plants, and their levels are comparatively low in dried goji berries (Zheng, Oellig, Zhu, et al., 2025). Goji berry FuFAs have recently attracted attention because of their crucial role as precursors of the key aroma compound, 3-methyl-2,4-nonadienone (Zheng, Oellig, Zhu, et al., 2025). The same study also reported the detection of the rare compound 9-(5-pentylfuran-2-yl)-nonanoic acid (9F5) in dried goji berries. These observations suggest that the primary significance of goji berry fatty acids may lie in determining fruit quality and sensory characteristics, whereas their direct therapeutic contribution requires further quantitative and clinical investigation.

### 3.4 Mechanistic integration and signaling pathway analysis

Rather than acting through a single molecular target, the biological effects of goji berry bioactive compounds arise from the coordinated modulation of multiple interconnected signaling pathways involved in inflammation, oxidative stress, apoptosis, mitochondrial function, metabolism, fibrosis, and immune regulation. Notably, different classes of phytochemicals, including polysaccharides, flavonoids, phenolic acids, carotenoids, coumarins, and phenylamides, often converge on common molecular pathways, despite their structural diversity (Figure 2). This convergence provides a mechanistic explanation for the diverse pharmacological activities described (Table 3B). Furthermore, the mechanisms of action differ according to the bioavailability of the compound. Low-bioavailability polysaccharides primarily exert indirect effects through gut microbiota-derived metabolites, whereas relatively bioavailable flavonoids, phenolic acids, and carotenoids directly modulate intracellular signaling cascades following absorption. Among these pathways, NF-κB is the most consistently regulated inflammatory signaling pathway. Numerous bioactive compounds suppress NF-κB activation, either directly or through the upstream modulation of MAPK, PI3K/AKT, and STAT signaling, resulting in the reduced production of pro-inflammatory mediators, including TNF-α, IL-1β, IL-6, COX-2, and iNOS. Consequently, NF-κB inhibition appears to be a common mechanistic denominator underlying the anti-inflammatory, anticancer, and tissue-protective effects of goji berry phytochemicals.

**Table 3B:** Mechanistic integration of major goji berry bioactive compounds.

**Bioactive compounds**	**Experimental model**	**Experimental dose**	**Bioavailability consideration**	**Signaling pathway**	**Level of evidence**	**References**
Berberine + Rutin	Murine rheumatoid arthritis	500 mg	Berberine poor oral bioavailability; rutin limited absorption	GSK3β/STAT/AKT/MAPK/NF-κB	Animal	(Sharma et al., 2022; Ai et al., 2021)
Neohesperidin	Mouse colorectal cancer	50 & 100 mg/kg of body weight	Poor bioavailability, metabolized extensively	NF-κB/p65, ERK/p38 MAPK	Animal	(Akhter et al., 2024; Cao et al., 2025)
Chlorogenic acid	LPS-induced neuroinflammation in HGF-1 cell line	25 & 50 μM	Poor absorption	NF-κB and PI3K/MAPK	*In vitro*	(Gonthier et al., 2003; Park & Yoon, 2022)
Catechin, *p*-Coumaric acid, Naringenin	High-fat diet male Wistar rats	5000 mg/kg of body weight	Faster absorption	NF-κB, TGF-β/Smad	Animal	(Banerjee et al., 2025; Pei et al., 2016)
Benzoic acid derivatives	Breast cancer cells	30 μM	Low bioavailability	NF-κB, STAT3, SMAD2, CREB, AKT	*In vitro*	(Fortin et al., 2025; Grgić et al., 2020)
Zeaxanthin	UVB-induced photodamage mice	0.45 mg/mL	Poor bioavailability	Nrf2/HO-1/NQO1	Animal	(Kotagiri et al., 2022; Zhu et al., 2025)

In a murine model of rheumatoid arthritis (RA), *Berberis lycium* fruit extract, containing berberine and rutin, attenuated complete Freund's adjuvant (CFA)-induced arthritis by suppressing inflammatory mediators and modulating the GSK3β/STAT/Akt/MAPK/NF-κB signaling pathways (Sharma et al., 2022). Neohesperidin inhibited macrophage infiltration and decreased TNF-α, IL-1β, IL-6, and COX-2 expression, exerting anticancer effects by targeting the NF-κB/p65 and ERK/p38 MAPK pathways in an azoxymethane/dextran sulfate sodium-induced mouse model of colorectal cancer (Cao et al., 2025). Chlorogenic acid (30 mg/kg/day) reduced inflammatory mediators (TNF-α, IFN-γ, COX-2, and iNOS) by targeting PI3K/AKT/NF-κB signaling, thereby decreasing LPS leakage, neurotoxicity, and alleviating LPS-induced pyroptosis (Lai et al., 2025). Catechin, *p*-coumaric acid, and naringenin inhibited the NF-kB and TGF-β/Smad pathways, improving kidney function, modulating oxidative stress, and reducing inflammatory cytokines, including TGF-β and collagen, in rats fed a high lipid diet (Banerjee et al., 2025). Benzoic acid derivatives inhibit EMT transcriptional factors, including NF-κB, STAT3, SMAD2, CREB, and AKT, thereby inhibiting EMT initiation or maintenance and exerting anticancer effects in breast cancer cells (Fortin et al., 2025). In addition to suppressing inflammatory signaling, goji berry phytochemicals consistently activate endogenous antioxidant defense pathways. Among these, the Nrf2 pathway serves as the principal regulator of cellular redox homeostasis by inducing antioxidant enzymes, including HO-1, NQO1, SOD, and CAT. Activation of Nrf2 complements the inhibition of NF-κB, highlighting the coordinated regulation of oxidative stress and inflammation rather than independent signaling events.

Zhu et al. (2025) reported that zeaxanthin supplementation decreased visible skin injury, epidermal thickening, collagen degradation, and wrinkle formation by enhancing antioxidant defense through the activation of the Nrf2 signaling pathway, which consequently leads to the activation of endogenous antioxidant enzymes such as HO-1, NQO1, SOD, and CAT against UVB-induced photodamage. Carotenoids play a crucial role in enhancing drought resistance in higher plants via the LbNAM2-LbZDS regulatory module (Ma, Liang, et al., 2025). Although this mechanism is plant-specific and not directly related to human therapeutic activity, it highlights the diverse biological functions of carotenoids in *Lycium* species. In addition to inflammation and oxidative stress, several phytochemicals regulate apoptosis, fibrosis, and cellular metabolism via the PI3K/AKT, TGF-β/Smad, and insulin/IGF-1 signaling pathways. These pathways influence cell proliferation, extracellular matrix remodeling, mitochondrial homeostasis, and longevity, thereby contributing to the reported anticancer, antifibrotic, anti-aging, and metabolic effects of goji berry constituents.

*Lycium ruthenicum* Murr. (black goji berry) is rich in caffeoyl-spermidine derivatives (caffeoyl-SPDs) with anti-aging and antioxidant potential. Network pharmacology analysis revealed the role of the insulin/IGF-1 signaling (IIS) pathway via daf-2 downregulation and daf-16 upregulation (Shen et al., 2026). Collectively, the available evidence suggests that goji berry bioactive compounds do not function through isolated signaling pathways but instead regulate an interconnected signaling network centered on NF-κB, MAPK, PI3K/AKT, Nrf2, STAT, TGF-β/Smad, and insulin/IGF-1 pathways. Crosstalk among these pathways provides a mechanistic basis for the simultaneous attenuation of inflammation, oxidative stress, apoptosis, fibrosis, and metabolic dysfunction in diverse disease models. However, the relative contributions of direct phytochemical–target interactions and indirect microbiota-mediated signaling remain poorly understood, particularly for poorly absorbed polysaccharides. Future studies integrating transcriptomics, proteomics, metabolomics, microbiome profiling, and pharmacokinetic analyses are required to establish causal pathway interactions and identify the principal molecular mediators responsible for the therapeutic effects of goji berry phytochemicals.

## 4 Food processing technologies and their impact on goji berry bioactivity

Dried fruit is the most common commercial form of goji berries, primarily aimed at extending self-life and facilitating export. Several drying methods are commonly practiced, such as conventional drying, in which fruits are shade-dried until their skin wrinkles and then exposed to sunlight, and convective drying, which is widely used in the food processing industry, where drying occurs in the presence of hot air (Radojčin et al., 2021). However, drying goji berries is slow and energy-intensive due to their thick waxy skin. Prolonged exposure to high temperatures can degrade bioactive components; therefore, various physical and chemical pretreatments have been explored to shorten the drying time and maintain the stability of phytochemicals in goji berries. Pretreatment methods, such as osmotic dehydration combined with pulsed electric field dehydration, have been found to decrease drying time while maintaining color, texture, and bioactive phytochemicals (Abshar et al., 2025). Recent studies have highlighted the use of advanced processing technologies, such as freeze–drying, vacuum drying, microwave-assisted drying, and infrared drying, to better preserve thermolabile compounds in goji berries, including carotenoids, polysaccharides, and phenolic compounds (Jian et al., 2025; Turan et al., 2023). Freeze-drying, in particular, has been reported to retain higher levels of *L. barbarum* polysaccharides, zeaxanthin, and antioxidant capacity than conventional hot-air drying methods. Table 4 shows the effects of food processing technologies on the bioactive compounds present in goji berries. However, the reported energy consumption for freeze-drying ranges from 600 to 1000 kWh per kg of water removed, which is significantly higher than that of spray drying or conventional hot-air drying (Roy & Rathod, 2024). Comparative investigations have suggested that freeze-drying is the best alternative method for preserving phenolic compounds in goji berry fruit; however, it has a long drying time, high cost, and limited scalability (Du et al., 2026; Turan et al., 2023). Different drying methods, including pulsed vacuum drying and hot wind drying, strongly influence browning reactions and shelf-life behavior, which are critical for consumer acceptance (Zhang, Shu, et al., 2025).

**Table 4:** Influence of food processing technologies on goji berry bioactive compounds.

**S. No**	**Processing technology**	**Key operating conditions**	**Drying time**	**Bioactive retention**	**Sensory & quality impact**	**Key bioactives affected**	**References**
1.	Hot air drying	50°C heated air circulation	Long drying time (1330 min)	Reduce heat-sensitive compounds due to prolonged exposure to heat	Color darkening and texture shrinkage	Vitamin C, carotenoids, polyphenols	(Huang et al., 2025; Turan et al., 2023)
2.	Vacuum microwaves combine with vacuum pulsation drying (VMD)	Microwave radiation (2W/g)	Rapid drying (15 – 60 min)	Moderate preservation of bioactives due to shorter drying time	Elliptical structure with smoother peel and irregular pores	Polysaccharides, flavonoids	(Jian et al., 2025**;**Tang et al., 2023)
3.	Freeze drying	Sublimation at low temperature (≤ −40 °C) under vacuum	Moderate drying time (5h)	Preserves most bioactive compounds and structural integrity	Best preservation of color, flavor, and structure	Polysaccharides, carotenoids, phenolics	(Jian et al., 2025)
4.	Vacuum drying	Reduced pressure (10–30 kPa) at moderate temperature	Moderate drying time (6–12 h); moderate throughput	Protects thermolabile compounds due to lower temperature	Better color and flavor retention compared to hot air drying	Carotenoids, vitamins, polysaccharides	(Zhang, Shu, et al., 2025)
5.	Pulsed electric field	High-frequency pulses (1kHz to 1MHz)	Used as pretreatment; reduces drying time	Enhances extraction and bioavailability of phytochemicals	Minimal damage and color retention	Polyphenols, flavonoids	(Dermesonlouoglou et al., 2018)
6.	High-pressure processing	Hydrostatic pressure (300–500 MPa)	Non-drying preservation method	Maintains bioactive integrity and enhances extractability	Minimal changes in flavor and nutrient content	Polysaccharides, antioxidants	(Hu et al., 2022)
7.	Ultrasound-Assisted Processing	Frequency (20 KHz)	Reduces drying time	Improves release and extraction of bioactive compounds	Minimal shrinkage rate	Phenolic compounds, polysaccharides	(Ni et al., 2020; Teixeira et al., 2024)
8.	Fermentation	Microbial metabolism modifies phytochemicals	Processing time (48 h)	Can increase antioxidant activity and bioavailability	Change in sweetness and sourness increased after fermentation	Phenolics, flavonoids, polysaccharides	(Duan et al., 2023)

Interestingly, experimental findings have shown that furan fatty acids are converted to volatile aroma compounds, such as 3-methyl-2, 4-nonadienone, in goji berries via an oxidative degradation process. Their conversion is strongly influenced by drying, particularly hot-air drying (temperature and oxygen), compared to the natural drying process. Therefore, drying conditions play a vital role in regulating the aroma development of goji berries (Zheng, Oellig, Zhu, et al., 2025). Recent evidence has shown that drying drives the conversion of fatty acid precursors into aroma compounds, and the same study identified 46 aroma compounds in goji berries, including major volatiles such as (E)-β-damascenone. During hot-air drying at 50 °C, furan fatty acids and lipid-derived volatiles were significantly reduced, indicating their conversion (Zheng, Oellig, Zhu, et al., 2025). Current evidence has analyzed flavor compounds in dried goji berries from different origins (Zhongning, Ningxia; Wuwei, Gansu; Nuomuhong, Qinghai) and drying methods. These studies showed that hot-air drying and natural sun drying produce distinct flavor characteristics owing to differences in the processes, including lipid oxidation and metabolite transformation pathways. Geographical origin also affects the metabolite composition and leads to variations in aroma (Dai et al., 2026).

Products developed using goji berry extracts or whole fruits exhibit high nutritional and antioxidant capacities. White chocolate supplemented with prebiotics and goji berry extracts exhibited excellent sensory characteristics, such as good odor and taste. Additionally, incorporating goji berries into foods extends their shelf life and prevents spoilage (Vidović et al., 2022). For example, the addition of goji berry extract to soybean oil prevents oxidative degradation, suggesting that organic extracts can be used as natural antioxidants in food products. The addition of goji berry extracts to minced catfish, sausages, horse meat, and beef effectively inhibited protein oxidation and lipolysis, increased phenolic content, and maintained an appealing texture, taste, tenderness, and pleasant aroma (Vidović et al., 2022). Emerging food processing techniques, such as high-pressure processing, pulsed electric fields, and ultrasound-assisted extraction, have also been explored to enhance the release and stability of bioactive compounds from goji berries while minimizing thermal degradation (Yildiz-Ozturk, 2025). These non-thermal technologies improve the extraction efficiency of polysaccharides and carotenoids and help maintain the natural color and antioxidant activity of processed products.

Instant gruels and rice-based extrudates fortified with dried goji berries contained higher levels of phenolic acids, zeaxanthin dipalmitate, and rutin, contributing to their enhanced antioxidant properties. Goji berries prolong the lifetime of lactic acid bacteria (LAB) in yogurt, maintaining probiotic activity during shipping and storage. During cheese ripening, the enzyme rennet breaks down the primary milk protein, casein, into small peptides. Some of these peptides have an angiotensin-converting enzyme (ACE) inhibitory effect, which decreases blood pressure when consumed in large amounts. Interestingly, cheese ripened and enriched with goji berry extracts showed decreased ACE inhibitory activity, which is due to bioactive peptides generated through lactic acid bacteria–mediated proteolysis of milk proteins and added substrates (e.g., fish collagen). These peptides inhibit ACE and consequently reduce the conversion of angiotensin I to angiotensin II, thereby leading to antihypertensive effects (Shori et al., 2021). This observation may reflect the interactions between goji berry phytochemicals and the dairy matrix, which influence the formation or activity of ACE-inhibitory peptides during fermentation, highlighting the importance of food matrix effects on the functional properties of goji berry-based products. Fermentation technologies have recently gained attention for improving the functional properties of goji berries. Fermentation with probiotic strains such as *Lactobacillus plantarum* and *L. rhamnosus* enhances polyphenol bioavailability and antioxidant activity while improving the flavor and digestibility of goji berry-based beverages and fermented foods (Kamonsuwan et al., 2024).

Prebiotic chocolates fortified with goji berries exhibited changes in aroma, texture, hardness, and bitterness, highlighting the need to optimize the sensory attributes in the development of functional probiotic chocolates. The incorporation of goji berry by-products (10, 20, 30, and 40 g/100 g) to replace wheat flour and develop bakery products, such as muffins and cookies, provides nutritional enhancement but simultaneously introduces significant sensory challenges. Sensory evaluation showed that consumer acceptability was dose-dependent, with acceptable incorporation levels limited to approximately ≤20% in muffins and ≤10% in cookies, whereas higher levels affected the overall quality (Bora et al., 2019). In particular, increasing the level of goji berry by-products decreased muffin firmness, cookie hardness, and fracturability (Bora et al., 2019). Recent research has also highlighted the sustainable utilization of goji berry processing residues (pomace and peel) as functional ingredients rich in dietary fiber and phenolics, which can be incorporated into bakery products, snack bars, and nutraceutical powders to improve their nutritional quality and reduce food processing waste.(Yu et al., 2023)

## 5 Role of goji berries in functional food product development

**Figure 3:**
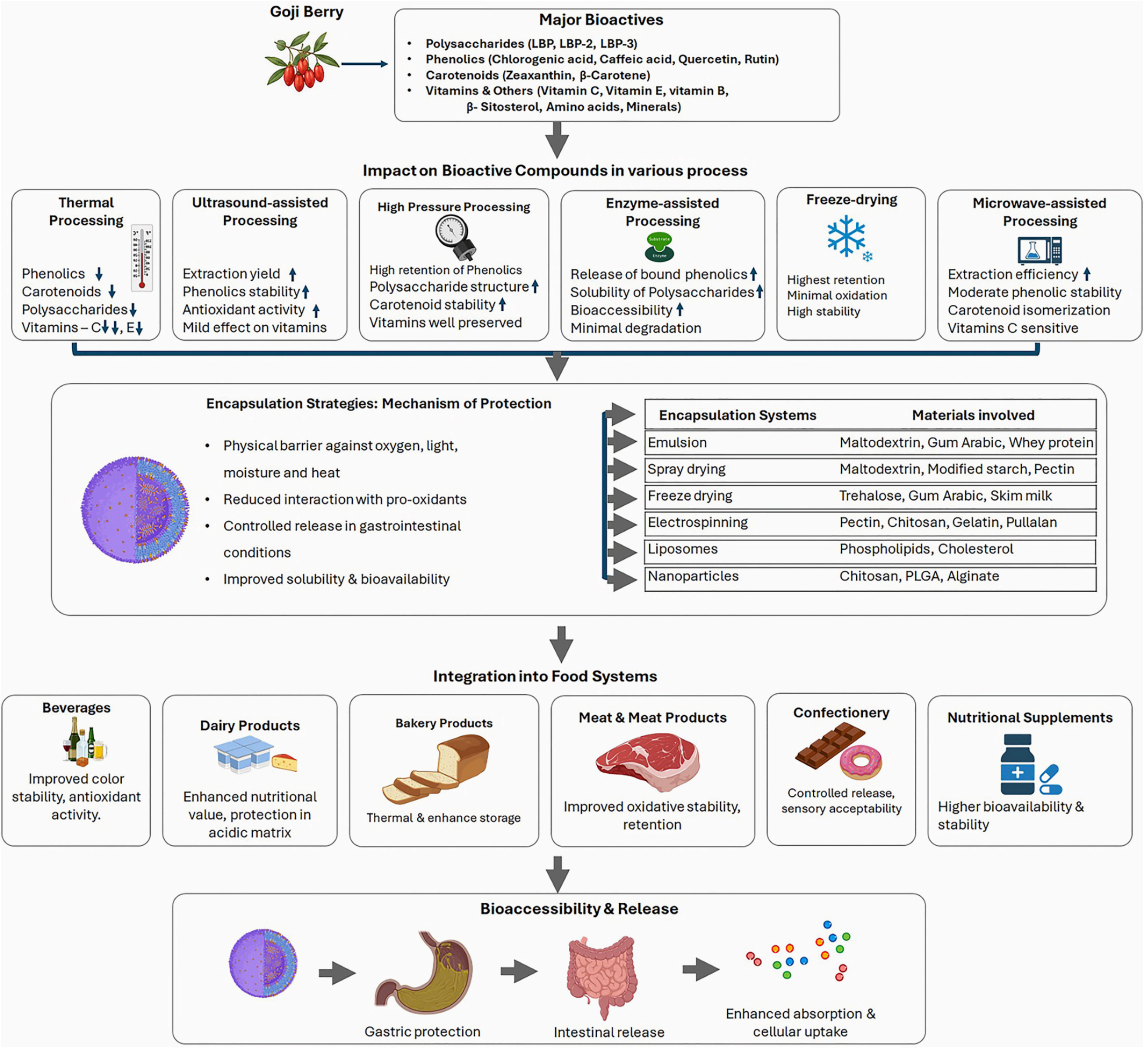
Technological integration of goji berry bioactives into food systems. Processing and encapsulation strategies enhance the stability, bioavailability, and functional performance of goji bioactives across diverse food applications.

Goji berries have been valued as both functional foods and traditional medicines by Asians for centuries. In recent years, the food industry has started using goji berries not only for their nutritional value but also for their enriched phytochemical content, such as anthocyanins, polyphenols, and flavonoids (Jurikova et al., 2025). Positioned within the “food-medicine homology” market, goji berries have been incorporated into various food products as functional ingredients, preservatives, and natural colorants (Lu et al., 2025). Figure 3 illustrates the application of goji berries and their bioactive compounds in various food systems. Encapsulation technologies have been widely employed to enhance the stability, bioavailability, and controlled release of key bioactive constituents such as *L. barbarum* polysaccharides, carotenoids, and phenolics (Păvăloiu et al., 2021). These encapsulated forms, along with the direct incorporation of goji fruits, have been successfully applied to diverse product categories, including confectionery, beverages, dairy formulations, and meat products. Such applications not only improve the nutritional and functional value of foods, but also extend the shelf life of products, mask undesirable flavors, and promote consumer acceptance. Recent advancements in food processing and encapsulation technologies have led to the development of a wide range of goji berry-fortified products. However, recent studies on goji berry extract encapsulation (e.g., nanoliposomes) have reported only moderate encapsulation efficiencies using microfluidics (∼46–49%), highlighting the limitations of effectively entrapping complex mixtures of bioactives (Peixoto et al., 2025). Moreover, in this section, we highlight the various applications of goji berries in functional food product development.

### 5.1 Application in bakery products

Wheat bread fortified with 10–30% goji berry powder exhibited reduced hardness, with 20% substitution providing the optimal texture and highest consumer acceptance, while also enhancing water absorption and gelatinization temperature (Hashemi et al., 2024). The incorporation of fresh goji berry pulp puree into bread modified its appearance without adversely affecting its flavor or taste and enhanced its inhibitory activities against lipase, α-amylase, and α-glucosidase, enzymes associated with metabolic syndrome (Sicari et al., 2023). Bread enriched with 50% and 70% goji puree showed an enhanced total phenolic content (42.07 mg gallic acid equivalent/100 g bread) and ABTS radical scavenging activity (833.48 μmol Trolox/100 g bread), with the highest potential observed at the 50% level (Loizzo et al., 2021). Hashemi et al. (2024) investigated the effects of incorporating 10, 15, 20, 25, and 30% goji berries into wheat bread. They found that approximately 20% goji berry content enhanced the specific volume and texture of the bread. However, excessive fiber content disrupts the gluten network, leading to reduced gas retention, inferior crumb structure, and decreased product quality.

The gel-forming ability of goji polysaccharides compensated for gluten weakening; however, this effect diminished at higher polysaccharide concentrations. Similarly, the acceptability of the sensory properties was enhanced up to the optimal level but reduced at higher concentrations owing to alterations in texture, color, and flavor. Thus, the successful incorporation of goji berries into bread requires optimization of the substitution level, adjustment of the water content, and modification of the mixing and fermentation conditions. Enriched energy bars developed using goji berries (12%) as one of the ingredients with fiber showed improvements in nutritional, physicochemical, antioxidant, and sensory qualities (Saravanan et al., 2026). Noreen et al. (2025) reported that goji berry products are used in muffins and cookies to enhance their texture, nutrient content, and cost-effectiveness. Recent studies have demonstrated that incorporating goji berry powder into gluten-free bakery formulations significantly improves dietary fiber and carotenoid levels while maintaining acceptable sensory properties, making them suitable for functional gluten-free products (Hashemi et al., 2024). The addition of goji berry extracts to bakery matrices has also been shown to retard lipid oxidation and extend shelf life owing to the presence of phenolics and carotenoids, thereby improving product stability during storage (Vidović et al., 2022). Figure 3 illustrates the application of goji berries in various bakery products.

### 5.2 Application in dairy products

Pectin extracted from goji berries exhibits a high extraction yield under optimal conditions (pH 2.2, 76 °C, and 80 min) and serves as a functional ingredient in yogurt, acting as a natural thickener while improving its nutritional and sensory properties (Geng et al., 2024). Anthocyanins extracted from goji berries (1 and 2 g/100 mL) have also been incorporated into yogurt and fermented milk, where they demonstrated high stability and significantly enhanced antioxidant capacity through increased DPPH radical-scavenging activity. Moreover, yogurt fortified with black goji berry anthocyanin extract exhibited significantly greater reducing power (p < 0.05) than yogurts containing sweet potato colorant or the control formulation (Gamage et al., 2024).

Goji berry supplementation further improves the functional quality of probiotic yogurt by enhancing the survival of *Lactobacillus acidophilus* and *Bifidobacterium lactis*, increasing total phenolic content, and strengthening antioxidant activity through DPPH radical scavenging (79.14% and 91.35%, respectively). These improvements were accompanied by increased viscosity and water-holding capacity, without adversely affecting sensory acceptability (Hacıoğlu & Ünal, 2026). Similarly, the incorporation of 1.5% *L. barbarum* into fermented milk drinks enhanced the health-promoting effects of probiotics, improved taste and nutritional value, and functioned as a natural stabilizer, making the product suitable for consumers of all ages (Slyvka et al., 2022).

Fruit enrichment has also been recommended to maintain the therapeutic levels of *Lactobacillus bulgaricus* and *Streptococcus thermophilus* in yogurt products. Yogurt supplemented with 7% goji berries, with or without honey, maintained the viability of lactic acid bacteria (LAB), promoted pH reduction (<4.0), and improved microbial stability throughout storage, thereby enhancing the functional value of the product (Rotar et al., 2014). Similarly, dairy products enriched with goji berries contained higher concentrations of polyphenols, and yogurt fortified with 6.0 ± 0.01% goji berries exhibited the greatest antioxidant capacity, with a DPPH radical scavenging activity of 63.30 ± 1.42%, primarily attributed to anthocyanins (Taneva & Zlatev, 2020).

The incorporation of goji berries (2.5–10%, w/v) into fermented dairy systems enhanced probiotic viability, stimulated lactic acid production, and reduced residual sugar content without compromising antioxidant activity, thereby improving the overall functional quality of the products (Kamonsuwan et al., 2024). Recent studies have demonstrated that triple-probiotic-fermented goji beverages restore gut microbiota homeostasis and represent promising probiotic-based functional foods (Ren et al., 2025). Yogurt fortified with goji berry extracts exhibited enhanced antioxidant capacity and an attractive purple color, improving consumer appeal. Similarly, wines and beers produced with goji berries showed increased antioxidant activity and superior sensory characteristics (Ducruet et al., 2017; Gamage et al., 2024). Zhang et al. (2023) developed goji berry juice fermented with *Lactobacillus rhamnosus*, which exhibited enhanced functional properties and antioxidant activity.

Goat milk powders enriched with lyophilized fruit extracts of *L. ruthenicum* and *L. barbarum* juice demonstrated enhanced antioxidant activities, including ABTS radical cation scavenging activity and ferrous ion-chelating capacity (FCC), while dose-dependently promoting the growth of probiotic strains, suggesting their potential as prebiotic supplements and functional food ingredients (Milinčić, Kostić, et al., 2024). Furthermore, goji berry polysaccharides incorporated into fermented dairy systems acted as natural prebiotic substrates, stimulating the proliferation of beneficial lactic acid bacteria and improving gut health-related functional properties (Skenderidis et al., 2019). Recent studies have also reported that microencapsulated goji berry extracts can be incorporated into yogurt and fermented milk to improve the stability of carotenoids and polyphenols during refrigerated storage, thereby enhancing the nutraceutical value of dairy products (Gruskiene et al., 2021; Skenderidis et al., 2019). Figure 3 summarizes the applications of goji berries in dairy products.

### 5.3 Application in beverages

Fermentation of *Lycium barbarum* pulp with Tibetan kefir grains enhanced the antioxidant activity of its polysaccharides by increasing the contents of arabinose, galactose, and uronic acids, highlighting its potential as a functional food ingredient with improved bioactivity (Qi et al., 2024). These findings provide a theoretical basis for optimizing the fermentation process and improving the flavor quality of Ningxia goji berry wine. Five important microorganisms associated with flavor compounds and 25 core microbes were linked to the non-volatile metabolites responsible for flavor formation (Peng, Huang, et al., 2025). In fermented black goji berry beverages, calcium-alginate encapsulation combined with carrageenan, agar, and gelatin enhanced the survival of *Lactobacillus rhamnosus* and stabilized phenolic content (Chusak et al., 2025). Milkshakes enriched with *L. barbarum* berries from Ningxia and their aqueous extracts showed significant antioxidant potential (radical-scavenging capacity using the DPPH assay) and increased phenolic content, including gallic acid, procyanidin B2, and catechin (Plucińska et al., 2022). During 21 days of refrigerated storage, yogurt supplemented with goji berries and/or fish collagen improved protein degradation over time, with slightly affected sensory properties (Shori et al., 2021). Functional beverages formulated with goji berry juice or extracts have gained increasing attention because of their high levels of polysaccharides, carotenoids, and phenolic compounds, which contribute to antioxidant capacity (DPPH radical scavenging, ABTS radical cation decolorization, ferric reducing antioxidant power, and oxygen radical absorbance capacity) and improved sensory profiles (Vidović et al., 2022; You, 2025). Additionally, the development of fermented probiotic drinks based on goji berry substrates has demonstrated enhanced bioavailability of phenolic compounds and improved stability of bioactive components during storage (Sun, Jiang, et al., 2025). Figure 3 depicts the incorporation of goji berries into beverages. Goji berry extracts and powders are used in kombucha, smoothies, energy boosters, and infusion teas, enhancing antioxidant capacity and supporting gut microbiota (Vidović et al., 2022). Their application in fermented products such as beer, yogurt, vinegar, and wine is also of significant value.

### 5.4 Application in snack and confectionery industry

Functional prebiotic white chocolate fortified with goji berries exhibited reduced aroma intensity and increased bitterness compared to the control. Despite these sensory changes, consumer acceptance remained moderate, with overall acceptance scores exceeding 6 on a 9-point hedonic scale. Partial least squares (PLS) regression analysis further indicated that cream color and cocoa butter flavor were the sensory attributes most strongly associated with consumer acceptance of functional white chocolate (Morais Ferreira et al., 2017). Goji berry fruit skin made from *L. barbarum* exhibited effective physicochemical and nutritional properties, with pH in the range of 4.96–4.99, increased dry matter, reduced water activity (0.37–0.38), and ultimately good shelf stability. Its texture characteristics include hardness, chewability, and stickiness, with acceptable color values and sensory properties (Maraş & Erdoğan, 2023). Sahu et al. (2024) reported that goji berries are extensively used as functional ingredients in confectionery and bakery products, including jams and jellies, with good color and sweetness properties. In foods such as extrudates, muffins, and cookies made using goji berry powder, the antioxidant compounds, phenolics, and sensory qualities, including flavor and acceptability, remain favorable. Cardoso et al. (2023) reviewed the use of goji berries in muffins, cookies, breads, tortillas, and snacks and highlighted the importance of total phenolic, protein, and dietary fiber contents, which also exhibited good sensory characteristics with consumer acceptance. Recent product development studies have indicated that incorporating goji berry powder into extruded snack formulations increases the total phenolic content, dietary fiber, and antioxidant capacity while maintaining crispness and consumer acceptability (Cardoso et al., 2023). Furthermore, goji berry extracts are increasingly utilized as natural colorants and flavor enhancers in confectionery products, offering an alternative to synthetic additives while improving the functional and nutritional profiles of snacks (Shunkai et al., 2025). Figure 3 highlights the diverse applications of goji berries in the snack and confectionery industries. Dried goji berries are widely used in tea brewing, snacks, baked products, and trail mixes, providing natural sweetness, antioxidants, and micronutrients such as vitamins B and C and minerals (Vidović et al., 2022). Their distinct applications are attributed to their high anthocyanin content, derived from petunidin, delphinidin, malvidin, and cyanidin. Anthocyanin pigments are sensitive to pH; they are pink or red under acidic conditions and blue or purple under alkaline conditions (Zhao, Gao, et al., 2023). The pH sensitivity of anthocyanin pigments has been exploited in the food industry for beverages, bakery items, and confectioneries as a natural alternative to synthetic colorants (Lakshmikanthan et al., 2024). Owing to their wide range of bioactive ingredients, such as polyphenols, polysaccharides, flavonoids, and carotenoids, goji berries are also used as supplements to boost immunity, protect eyesight, and support anti-aging. Furthermore, they are added to various dietary products, protein bars, and cereals to provide a natural appeal and functional activity (Qin et al., 2021). Their high sugar content and chewy texture make them suitable for bakery products, such as fillings, cookies, granola bars, and confections. Goji berry-based jellies and jams, rich in phenolic compounds, exhibit high antioxidant capacity and high scores for flavor, sweetness, and texture. Goji berry powders and extracts have also been incorporated into candies and chocolates, making them more appealing to health-conscious consumers seeking natural and functional ingredients (Jiang et al., 2021).

### 5.5 Application for food preservatives

Goji berry extracts, which are rich in phenolic acids and other bioactive compounds with antimicrobial properties, have attracted considerable attention as natural food preservatives. Anthocyanins isolated from goji berries have been incorporated into edible films, either alone or blended with polymer matrices, to extend the freshness of dairy, seafood, and red meat products. Owing to their pH-sensitive color changes, these anthocyanins have also been utilized as intelligent indicators of food spoilage, providing sustainable approaches for active food packaging and quality-monitoring. In addition, goji berry extracts function as natural antioxidants by delaying lipid peroxidation and preserving meat freshness (Menchetti et al., 2020). Recent studies have demonstrated the successful incorporation of goji berry powder and extracts into plant-based dairy alternatives, gluten-free bakery products, and functional beverages, enhancing antioxidant activity, improving color stability, and increasing nutritional quality (Vidović et al., 2022). Advances in food processing have enabled the application of goji berry polysaccharides as natural stabilizers and texture-modifying agents in beverages, emulsions, and dairy formulations, highlighting their multifunctional role in modern food product development (Shi et al., 2024). Moreover, goji berry bioactive compounds are increasingly incorporated into active and biodegradable packaging materials, where their antioxidant and antimicrobial properties extend the shelf life of food while supporting the development of sustainable packaging technologies (Vidović et al., 2022).

## 6 Forms of goji berry used in nutraceutical and functional food products

Encapsulation technologies have been widely investigated to improve the stability, bioavailability, and functionality of goji berry bioactives in nutraceutical products. Table 5 presents the different forms of goji berry products, their processing methods, associated bioactive components, and their applications in nutraceuticals and functional foods. The encapsulation of goji berry extracts prepared using ultrasound-assisted extraction and spray-dried with polymers, such as alginate, pectin, and Eudragit E100, retained antioxidant potential and enhanced the stability and functional performance of bioactive compounds, suggesting promising applications in nutraceutical formulations (Teixeira et al., 2025). The incorporation of up to 20% goji berry powder into wheat bread dough maintained desirable dough properties and improved bread quality by increasing specific loaf volume (∼10%), reducing crumb hardness, and enhancing consumer sensory acceptance (Hashemi et al., 2024).

**Table 5:** Forms of goji berry products, processing methods, bioactive components, and their applications in nutraceuticals and functional foods.

**S. No**	**Goji berry products**	**Processing methods**	**Applications in Nutraceuticals and Functional Foods**	**Bioactive Components**	**Health Benefits**	**References**
1.	Dried goji berries	Fresh berries dehydrated using sun drying, hot-air drying, or freeze drying	Consumed directly as snacks, added to cereals, bakery products, salads, and teas	Polysaccharides (LBP), carotenoids, phenolic compounds, vitamins	Antioxidant, immune boosting, anti-aging effects	(Vidović et al., 2022)
2.	Goji berry powder	Freeze-dried or spray-dried berries ground into fine powder	Capsules, dietary supplements, smoothies, nutrition powders	Polyphenols, polysaccharides, carotenoids	Anti-inflammatory, antioxidant, immune modulation	(Shunkai et al., 2025; Vidović et al., 2022)
3.	Goji berry juice	Fresh berries processed into juice through crushing, filtration, and pasteurization	Functional beverages, energy drinks, health tonics	Carotenoids, vitamin C, flavonoids, polysaccharides	Antioxidant activity, improved metabolic health	(Ilić et al., 2025)
4.	Goji berry extract	Concentrated extract obtained using solvent extraction or enzymatic methods	Nutraceutical capsules, tablets, herbal supplements	*Lycium barbarum* polysaccharides (LBP), flavonoids	Neuroprotective, anti-diabetic, anti-cancer potential	(Qiang et al., 2023)
5.	Goji berry tea / Infusion	Dried berries infused in hot water	Herbal tea blends and functional beverages	Phenolics, flavonoids, polysaccharides	Digestive health, immune support	(Jurikova et al., 2025)
6.	Goji berry oil	Oil extracted from seeds using cold pressing or solvent extraction	Functional oils, cosmetic nutraceuticals	Essential fatty acids, carotenoids, tocopherols	Skin health, antioxidant protection	(Teixeira et al., 2023)
7.	Goji berry fermented products	Fermentation using probiotic bacteria or yeast	Functional beverages, probiotic drinks, fermented nutraceutical products	Modified phenolics, enhanced antioxidants	Gut microbiota modulation, improved bioavailability	(Kamonsuwan et al., 2024)
8.	Goji berry gummies / functional confectionery	Berry extracts incorporated into gelatin or plant-based matrices	Functional snacks and nutraceutical gummies	Carotenoids, vitamin C, polyphenols	Antioxidant supplementation and immune support	(Shunkai et al., 2025)
9.	Goji berry enriched foods	Goji berry powder or extract incorporated into foods	Yogurt, cereals, granola bars, bakery products, dairy products	Polyphenols, carotenoids, polysaccharides	Functional food enrichment with antioxidant benefits	(Shunkai et al., 2025; Vidović et al., 2022)

Administration of probiotic-fermented goji juice (20 mL/kg/day) for 30 days modulated inflammatory responses and enhanced antioxidant enzyme activities in the serum and colon of mice (Liu et al., 2021). Harvesting time also significantly influences the nutraceutical quality of goji berries by affecting the accumulation of bioactive compounds such as flavonoids, polyphenols, and carotenoids. Increased flavonoid content and antioxidant activity have been reported in the early stages of the fruiting season in berries cultivated in central Italy under different harvesting conditions, such as the beginning and late phases of the fruiting season (Poggioni et al., 2022). Similarly, Shi et al. (2024) highlighted that the nutritional composition of goji berries varies according to geographical origin, cultivation practices, and processing methods, emphasizing the need for further studies on the bioavailability, metabolism, and synergistic interactions of bioactive compounds in goji berries.

Recent developments in food and nutraceutical technology have focused on incorporating goji berry extracts into functional beverages, dairy products, snack bars, and dietary supplements to enhance their nutritional value and antioxidant capacity (Ma et al., 2023; Vidović et al., 2022). Advanced delivery systems, such as nanoemulsions and liposomal encapsulation, have been developed to improve the stability, controlled release, and bioavailability of carotenoids and polysaccharides from goji berries in functional foods and nutraceutical formulations (Peixoto et al., 2025; Shi et al., 2024). The encapsulation of goji berry extract into liposomes using microfluidic technology has been reported to enhance stability and delivery and increase antioxidant and anticancer activities (Peixoto et al., 2025). Fermentation of goji berries with probiotic microorganisms has recently emerged as an innovative approach to enhance the bioaccessibility of phenolic compounds and improve the sensory properties of functional food products (Hu et al., 2023). Fermentation of *Lycium barbarum*-derived goji juice using *Lacticaseibacillus rhamnosus* caused changes in both volatile and non-volatile metabolite profiles. Additionally, it causes a reduction in sugars, phenolic acids, aroma precursors, and volatile-related compounds (including carotenoid-derived volatiles), with an increase in lactic acid, leading to undesirable properties (An et al., 2024).

## 7 Safety, toxicology, and regulatory aspects

Goji berries are considered functional foods rich in antioxidants and nutrients that are beneficial for health and well-being. They alleviate oxidative stress and confer several health benefits, including the inhibition of free radical formation and decreased damage to biomolecules such as DNA, lipids, and proteins. Their health-promoting properties include anti-aging, hepatoprotective, anti-inflammatory, and antioxidant properties (Jiang et al., 2021; Ma et al., 2022). Traditionally, dried berries are cooked before consumption and are commonly used in Chinese soups, herbal teas, tinctures, wines, and juices. The recommended daily intake of goji berries ranges from approximately 14 to 20 g; however, this dosage may be insufficient to achieve certain therapeutic effects and may require a combination with other medicinal herbs to enhance efficacy (Kakici & Gezer, 2025). The common carotenoids present in goji berries include zeaxanthin dipalmitate, lutein, and zeaxanthin. A study on bioavailability showed that the bioavailability was in the order of zeaxanthin dipalmitate>zeaxanthin> lutein in differentiated Caco-2 cells (Juan et al., 2022). Preclinical investigations using *Drosophila* showed that LBP exhibited two-phase dose effects like 0-150 mg/kg and 450-100 mg/kg increased sperm motility, while at the dose–150-450 mg/kg and greater than 100 mg/kg, it inhibited sperm motility. However, in-depth studies on the constituents of goji berries and their effects on reproductive toxicity are essential to optimize their therapeutic potential (Yu et al., 2025). Additionally, goji berries elevate liver function, modulate CYP2C9 and CYP2E1 expression, and exert immunomodulatory effects on the immune system. The interactions between goji berries and prescribed medications have also been examined. Although goji berries are valuable crops rich in nutrients and bioactive compounds that promote health, their high sugar content makes them highly vulnerable to pests and diseases, particularly aphids and mites (Chen et al., 2023). Statistical analyses have shown that aphid infestations can reduce goji berry production by up to ¼, ultimately affecting food quality (Huang et al., 2021). Farmers have reported that the limited number of pesticides approved for use on goji berries is insufficient for pest control, thereby affecting production levels (Chen et al., 2023). Consequently, various fungicides and insecticides are employed to manage pests (fruit flies and scabs) and avoid severe financial losses (Chen et al., 2023). According to the European Food Safety Authority (EFSA (European Food Safety Authority), 2017), dimethoate residues in goji berries have been reported to exceed the European Union (EU) maximum residue limit (MRL). Therefore, the development and appropriate use of plant protection products specifically tailored for goji berry cultivation are essential to ensure effective pest management while minimizing pesticide residues (Chen et al., 2023). The FAO/WHO (2017) recommended expanding supervised field trials across diverse crop groups, including cereals, vegetables, and root and tuber crops, to improve the assessment and regulation of pesticide use in secondary crops. However, crop-specific regulatory measures for minor crops such as goji berries remain limited. Expanded pesticide registration and additional supervised field trials are therefore essential to establish appropriate maximum residue limits (MRLs), ensure consumer safety, and minimize concerns regarding pesticide residues (Chen et al., 2023).

## 8 Future perspectives

This review summarizes the nutritional and phytochemical composition of goji berries and their associated health benefits. Rich in polysaccharides, flavonoids, carotenoids, and essential nutrients, goji berries exhibit antioxidant, prebiotic, and functional properties that support gut health and overall well-being. Their diverse bioactive compounds have promoted their application in functional foods, beverages, nutraceuticals, and cosmetics as sustainable alternatives to synthetic additives. Despite substantial progress in elucidating the biological activities of goji berries, their successful translation into evidence-based functional foods and nutraceuticals remains limited by four major challenges: (i) poor and variable bioavailability of key phytochemicals, (ii) inadequate clinical validation, (iii) instability of bioactive compounds during processing and storage, and (iv) lack of standardized raw materials and quality control. Addressing these bottlenecks should be prioritized over expanding the number of reported pharmacological activities of goji berries. First, improving the bioavailability of goji berry phytochemicals remains the most critical research priority. Although numerous studies have demonstrated promising biological activities, the absorption, metabolism, tissue distribution, and excretion of many compounds, particularly polysaccharides and polyphenols, remain poorly understood. Future studies should combine pharmacokinetic analysis with metabolomics and gut microbiome profiling to identify the bioactive metabolites responsible for these physiological effects. In parallel, formulation strategies, such as nanoencapsulation, liposomes, and food-grade delivery systems, should be evaluated using standardized *in vivo* and human intervention studies to determine whether improved bioavailability translates into enhanced clinical efficacy. Second, stronger clinical evidence is required to validate the health benefits of goji berry consumption. Most available evidence is derived from *in vitro* experiments or animal models using purified compounds at pharmacological doses, which may not reflect habitual dietary intakes. Future research should prioritize well-designed randomized controlled trials that employ standardized goji berry preparations, physiologically relevant doses, and clinically meaningful outcome measures. Such studies should also evaluate long-term safety, dose–response relationships, and potential interactions with commonly prescribed medications. Third, preserving phytochemical stability during processing is essential for maintaining the nutritional and functional quality of goji berry products. Processing conditions, drying methods, storage, and food matrices can substantially influence the stability and bioaccessibility of polysaccharides, flavonoids, carotenoids, and other food bioactive compounds. Future studies should focus on optimizing processing technologies and delivery systems to maximize compound stability, while preserving sensory quality and consumer acceptability. Finally, the development of internationally accepted quality standards is essential for improving study reproducibility and facilitating regulatory approval. Standardizing plant materials, phytochemical compositions, extraction methods, and analytical procedures will reduce variability between studies and strengthen the scientific basis for health-related claims. Harmonized quality control protocols will also improve product consistency and consumer confidence in goji berry-based functional foods and nutraceuticals. Overall, this review provides a comprehensive overview of the nutritional composition, bioactive constituents, functional properties, processing, safety, and future perspectives of goji berries (*Lycium* spp.). Future research should move beyond simply identifying additional bioactive compounds and focus on resolving the key translational barriers that currently limit their clinical applications. Integrating pharmacokinetics, microbiome science, food technology, and well-designed clinical trials will establish the mechanistic and clinical evidence required to develop scientifically validated goji berry-based functional foods and nutraceuticals.

## 9 Conclusion

Goji berries are considered a highly promising class of functional ingredients, combining rich nutritional value with a broad spectrum of bioactive compounds, including phenolics, carotenoids, and essential fatty acids. These constituents underpin diverse health-promoting properties, particularly antioxidants, eye-protective, immunomodulatory, and anti-aging effects. Advancements in food processing and formulation have extended their application to bakery, dairy, beverage, and snack products. Moreover, maximizing their commercial and therapeutic potential will require standardized cultivation, efficient phytochemical characterization, optimized processing technologies, and well-structured clinical studies. With a combination of interdisciplinary research and innovation, goji berries are expected to play an increasingly significant role in the next generation of functional foods, nutraceuticals, and health products.

## CRediT authorship contribution statement

**Muthu Thiruvengadam:** Writing – original draft, Conceptualization. **Hee-Youn Chi:** Methodology, Data curation. **Ja-Min Lee:** Methodology, Formal analysis. **Yunwoo Park:** Visualization, Software. **Dagyeom Jeon:** Visualization, Resources. **Seung-Bin Lee:** Methodology. **Yong-Ik Jin:** Writing – review & editing, Validation. **Hae Won Jang:** Writing – review & editing, Validation. **Rekha Thiruvengadam:** Writing – review & editing. **Baskar Venkidasamy:** Writing – review & editing. **Seung-Hyun Kim:** Writing – review & editing, Supervision, Investigation, Funding acquisition, Conceptualization.

## Declaration of competing interest

The authors declare that they have no competing financial interests or personal relationships that could have influenced the work reported in this paper.

## Data Availability

No data was used for the research described in the article.
